# The epigenetic factor *Zrf1* regulates intestinal stem cell proliferation during midgut regeneration

**DOI:** 10.1371/journal.pgen.1011910

**Published:** 2025-10-27

**Authors:** Joshua Shing Shun Li, Ying Liu, Ah-Ram Kim, Mujeeb Qadiri, Jun Xu, Baolong Xia, Richard Binari, John M. Asara, Yanhui Hu, Norbert Perrimon

**Affiliations:** 1 Department of Genetics, Blavatnik Institute, Harvard Medical School, Boston, Massachusetts, United States of America; 2 Department of Medicine, Harvard Medical School, Boston, Massachusetts, United States of America; 3 Division of Signal Transduction, Beth Israel Deaconess Medical Center, Boston, Massachusetts, United States of America; 4 Howard Hughes Medical Institute, Boston, Massachusetts, United States of America; University of Helsinki: Helsingin Yliopisto, FINLAND

## Abstract

Stem cells are essential for tissue maintenance and regeneration, balancing self-renewal and differentiation to support homeostasis and repair. Through an RNAi screen in the *Drosophila* midgut, we identified the epigenetic factor Zrf1 as a critical regulator of intestinal stem cell (ISC) proliferation. Functional analyses reveal that Zrf1 integrates inputs from multiple signaling pathways and interacts with components of the RNA-induced silencing complex (RISC). Our findings suggest that Zrf1 is potentially a key chromatin regulator necessary for maintaining stem cell proliferation, enhancing our understanding of the molecular controls underlying stem cell function and chromatin-associated defects.

## Author summary

Tissue homeostasis, such as the constant renewal of the intestinal epithelium, relies on the precise regulation of adult stem cells. Understanding the molecular cues that govern stem cell proliferation is critical for regenerative medicine and for deciphering pathologies like cancer. Using the Drosophila midgut as a model, we identified the epigenetic factor Zrf1 as a key regulator of intestinal stem cell (ISC) proliferation. Functional analyses reveal that Zrf1 integrates inputs from multiple conserved signaling pathways, including EGFR, Notch, and Yorkie, to control the regenerative response. Mechanistically, Zrf1 physically associates with components of the RNA-induced silencing complex (RISC). However, its function is not in canonical RNAi, but in epigenetic regulation. Zrf1 modulates chromatin-based gene silencing, in part through genetic interactions with Polycomb group proteins, thereby influencing gene expression programs essential for proliferation. Our research uncovers a novel regulatory axis that links mitogenic signaling to the epigenetic state of stem cells, providing a deeper understanding of tissue regeneration and potentially new therapeutic targets for diseases characterized by deregulated stem cell activity.

## Introduction

Stem cells are fundamental to the maintenance and regeneration of tissues across multicellular organisms. These cells possess the remarkable ability to both self-renew and differentiate into various cell types, ensuring tissue homeostasis and repair. Understanding the molecular mechanisms that regulate stem cell behavior is crucial for advancing regenerative medicine and cancer therapy.

The *Drosophila* midgut is a well-established system for studying mechanisms that maintain stemness and stem cell differentiation [1,2]. The fly midgut, a tube lined by an epithelial monolayer, is primarily composed of secretory enteroendocrine cells (EEs) and absorptive enterocytes (ECs). Resident intestinal stem cells (ISCs) continuously divide to replenish these cells as the midgut responds to damage. Many of the major pathways that orchestrate this process in *Drosophila* ISCs, including Notch, JAK/STAT, Hippo, Insulin, EGF and Wnt pathways, have also been shown to regulate mammalian epithelial stem cells [3,4,5]. These pathways initiate transcriptional programs that chromatin remodelers then shape through epigenetic modifications. Epigenetic mechanisms and chromatin regulators are crucial for fine-tuning gene expression without altering the underlying DNA sequence, especially in response to stress or damage that necessitates tissue repair [6,7]. For example, PcG proteins (e.g., PRC2 complex: E(z), Su(z)12) maintain repression of developmental and proliferative genes under homeostatic conditions [8]. Upon injury, PcG-mediated repression is relieved, allowing activation of regenerative programs. Further, TrxG proteins, which counteract PcG, promote activation of genes required for proliferation and lineage commitment during regeneration. Increasingly, other studies in *Drosophila* have also highlighted the role of chromatin regulators in ISC proliferation and differentiation [9,6,7].

In a screen biased toward DNA binding proteins, we identified *CG10565* as a regulator of ISC proliferation in the adult *Drosophila* midgut. *CG10565* (hereafter as *Zrf1*) encodes the ortholog of Zuotin-related factor 1 (ZRF1), which has been shown to displace Polycomb-repressive complex 1 from chromatin to facilitate transcriptional activation [10]. Fly Zrf1 contains several functional domains, including a homeodomain-like region in the central portion of the protein and a C-terminal SANT domain, both potentially contributing to DNA or chromatin interactions. We found that Zrf1 interacts with multiple signaling pathways that regulate ISC proliferation and acts downstream of Myc, which has been shown to integrate proliferation cues from multiple signaling pathways [11]. By leveraging genetic tools, immunoprecipitation-mass spectrometry (IP-MS), and AlphaFold-Multimer (AFM) analyses, we present a comprehensive analysis of the role of Zrf1 in ISC proliferation and chromatin regulation. Our findings contribute to the broader context of how chromatin regulators influence stem cell biology and tissue regeneration.

## Results

### *Zrf1* is necessary for ISC proliferation during homeostasis and regeneration

**Fig 1:**
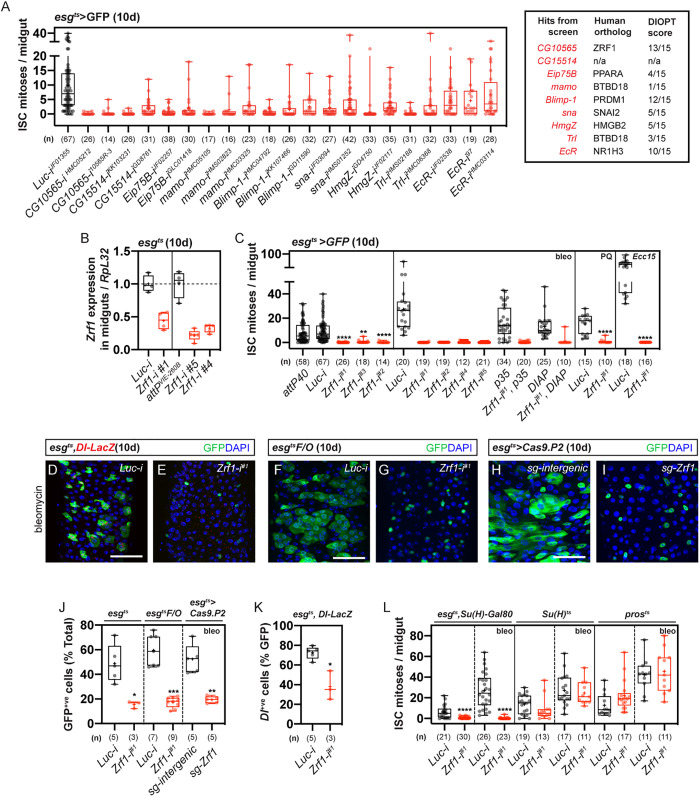
Genetic screen to identify *Zrf1* as a regulator of ISC proliferation during homeostasis and in response to damage. **(A)** Box plot showing ISC mitoses per midgut in *egt*^*ts*^* > GFP* flies after 10 days of RNAi induction targeting candidate genes enriched in intestinal progenitors. Hits from the screen are listed with their human orthologs and DIOPT scores. *Zrf1* RNAi lines (HMS00624, KK105682) led to reduced ISC mitosis compared to the control (*Luciferase, Luc*). Numbers below each box plot indicate the number of midguts analyzed. See also S1 Data Table **A. (B)** Quantification of *Zrf1* transcript levels in midguts after 10 days of *Zrf1* knockdown in midgut progenitors, showing reduced expression relative to the housekeeping gene *RpL32* compared to controls. See also S1 Data Table **B. (C)** Box plot showing ISC mitoses per midgut after *Zrf1* knockdown during homeostasis and following treatment with damage-inducing agents (bleomycin, paraquat, Ecc15). ISC mitoses are also shown following co-expression of anti-apoptotic factors (DIAP or p35). Numbers below each plot indicate the number of midguts analyzed. See also S1 Data Table **C. (D–H)** Confocal images of midguts from *egt*^*ts*^ flies treated with bleomycin. **(D)** Control (*Luciferase, Luc*). **(E)** *Zrf1* knockdown showing reduced ISC proliferation. **(F–G)** Lineage tracing using *egt*^*ts*^*F/O* system: **(F)** Control (*Luc*) and **(G)** *Zrf1* knockdown. **(H)** CRISPR/Cas9-mediated knockout of *Zrf1* (*egt*^*ts*^* > Cas9.P2*) showing similar reduction in ISC division. GFP marks progenitor cells; DAPI marks nuclei. Scale bar: 50 µm. See also S1 Data Table **D. (J)** Box plot quantifying the percentage of GFP+ cells from experiments after *Zrf1* knockdown and control (Luc) in midgut progenitors. **(K)** Quantification of Dl-LacZ positive cells (ISC) after midgut-progenitor-specific *Zrf1* knockdown in comparison to control. See also S1 Data Table **E. (L)** Box plots showing ISC mitoses per midgut after *Zrf1* downregulation in specific gut cell types: ISCs (*egt*^*ts*^* + Su(H)-Gal80*), enteroblasts (*Su(H)*^*ts*^) and enteroendocrine cells (*pros*^*ts*^). Midguts were treated with bleomycin. Numbers below each box plot indicate the number of midguts analyzed. See also S1 Data Table **F.** Box plots show median, quartiles, and range. Statistical significance determined by Student’s t-test; ns = not significant, *p < 0.05, **p < 0.01, ***p < 0.001 ****p < 0.0001.

To identify intrinsic factors essential for ISC proliferation, we performed an RNAi screen of candidate genes biased towards DNA-binding proteins enriched in intestinal progenitors [3,12,13]. Utilizing the ISC-specific gene expression driver *Escargot (esg)-Gal4* combined with the TARGET system (*tub-Gal80*^*ts*^) [14] and *UAS-GFP*, hereafter referred to as *egt*^*ts*^, we selectively drove *UAS-RNAi* in adult ISC/EBs. Progenitors were visualized using *UAS-GFP*, and anti-phospho-histone 3 (pH3) staining served as a marker for ISC mitosis. Among ~120 RNAi lines targeting ~73 genes, three independent RNAi transgenes targeting *CG10565 (*hereafter referred to as *Zrf1*) significantly reduced ISC proliferation during homeostasis and in response to damage-inducing diets (Figs S1Aand 1A-1C). *Zrf1* knockdown also resulted in fewer *Delta-LacZ*-positive and GFP-positive cells (Fig 1D, 1E, 1J and 1K). Lineage tracing using the *egt*^*ts*^*F/O* system [15] revealed that progenitors failed to divide following *Zrf1 RNAi* knockdown (Figs 1F, 1G, 1J, S1B and S1C)*.* CRISPR/Cas9-mediated knockout (*egt*^*ts*^* > Cas9.P2)* of *Zrf1* in ISC/EBs produced a similar phenotype (Fig 1H-1J). Importantly, the reduction in ISC proliferation was not a result of cell death since blocking apoptosis, by overexpressing either *DIAP* or *p35* in progenitors, could not rescue this defect (Fig 1C). The proliferation in response to damage in ISCs was unaffected when *Zrf1* was downregulated in adult enteroblasts (*Su(H)*^*ts*^) or in EEs (*pros*^*ts*^) (Fig 1L). Regeneration of ISCs was only impaired when *Zrf1* was knocked down specifically in ISCs (*egt*^*ts*^* + Su(H)-Gal80* or *Dl*^*ts*^) (Figs 1L and S1E). Altogether, these results indicate that *Zrf1* is cell-autonomously required for the proliferation of ISCs during homeostasis and regeneration.

### *Zrf1* promotes ISC proliferation in response to damage

**Fig 2:**
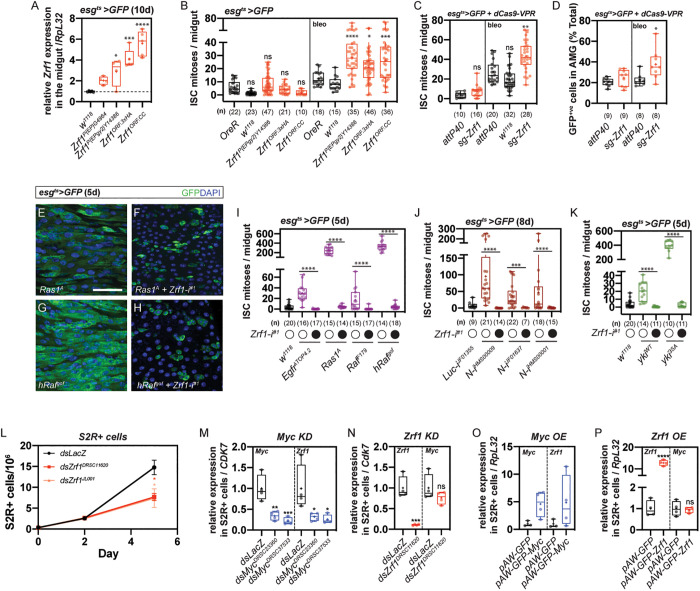
Zrf1 promotes ISC proliferation in response to damage. **(A)** Relative *Zrf1* expression levels in midguts from *egt*^*ts*^* > GFP* flies after 10 days of overexpression using different transgenes. Expression was measured relative to the housekeeping gene *RpL32*. Overexpression of *Zrf1* (EPgy2Y14386, ORF.3xHA, and ORF.CC) resulted in increased *Zrf1* transcript levels compared to the control (wild type, *w*^*1118*^). See also S1 Data Table **I. (B)** ISC mitoses per midgut in *egt*^*ts*^* > GFP* flies overexpressing *Zrf1* transgenes (EPgy2Y14386, ORF.3xHA, ORF.CC) during homeostasis and after bleomycin treatment (bleo). Number of midguts analyzed is shown below each box plot. See also S1 Data Table **J. (C)** Quantification of ISC mitoses in whole midguts of *egt*^*ts*^* > GFP* + *dCas9-VPR* flies, comparing *sg-Zrf1* to controls (*attP40, sg-intergenic*) under homeostasis and bleomycin treatment conditions. See also S1 Data Table **K. (D)** Quantification of GFP+ progenitor cells in the anterior midgut (AMG) of *egt*^*ts*^* > GFP* + *dCas9-VPR* flies, showing the effect of *sg-Zrf1* compared to *sg-intergenic* controls. See also S1 Data Table **L. (E–H)** Confocal images of ISC/EB progenitors in *egt*^*ts*^* > GFP* flies overexpressing fly *Ras1A* or human gain-of-function Raf (*hRaf*^*gof*^) alone or in combination with *Zrf1* knockdown. Knockdown of *Zrf1* rescued ISC overproliferation induced by activated Ras1 and hRaf. GFP marks progenitor cells; DAPI marks nuclei. Scale bar: 50 µm. **(I)** ISC mitoses per midgut after 5 days of ectopic activation of the EGFR/Ras/Raf signaling components (Egfr^TOP4.2^, Ras1A, Raf^F149^, hRaf^gof^) in *egt*^*ts*^* > GFP* flies with and without *Zrf1* knockdown (Zrf1HMS00624). See also S1 Data Table **M. (J)** ISC mitoses per midgut after 8 days of Notch knockdown in *egt*^*ts*^* > GFP* flies with and without *Zrf1* knockdown. See also S1 Data Table **N. (K)** ISC mitoses per midgut after 5 days of overexpressing wildtype *Yorkie* (*Yki*^*WT*^) or constitutively active *Yorkie* (*Yki*^*3SA*^) in *egt*^*ts*^* > GFP* flies with and without *Zrf1* knockdown. See also S1 Data Table **O. (L)** Growth curves of S2R+ cells over a 6-day period showing cell proliferation after dsRNA mediated knockdown of *Myc* (*dsMyc*), *Zrf1* (*dsZrf1*), and *LacZ* (*dsLacZ,* control). See also S1 Data Table **P. (M)** Relative expression of *Myc* and *Zrf1* in S2R+ cells following knockdown of *Myc*, showing that *Myc* knockdown reduces *Zrf1* transcript levels but not vice versa. See also S1 Data Table **Q. (N)** Relative expression of *Myc* and *Zrf1* in S2R+ cells following knockdown of *Zrf1*, indicating that *Zrf1* knockdown does not affect Myc expression. See also S1 Data Table **R. (O)** Relative expression of *Myc* and *Zrf1* following *Myc* overexpression (*pAWGFP-Myc*) in S2R+ cells, showing that *Myc* overexpression increases *Zrf1* transcript levels. See also S1 Data Table **S. (P)** Relative expression of *Myc* and *Zrf1* in S2R+ cells following *Zrf1* overexpression (*pAWGFP-Zrf1*), showing that *Zrf1* overexpression does not affect *Myc* transcript levels. See also S1 Data Table **T.** Box plots show median, quartiles, and range. Statistical significance determined by Student’s t-test; ns = not significant, *p < 0.05, **p < 0.01, ***p < 0.001 ****p < 0.0001.

To determine whether *Zrf1* is sufficient to promote ISC proliferation, we tested transgenes for effective *Zrf1* overexpression. We independently drove overexpression of four transgenes in ISC/EBs. These included two EP-like insertions, *P{EP}G4964* and *EP{EPgy2}Y14386*, which both consist of a *UAS* sequence inserted into the 5’ region upstream of the endogenous *Zrf1* gene as well as two *UAS-FlyORF* lines (*Zrf1*^*ORF.3xHA*^ and *Zrf1*^*ORF.CC*^). Overexpression of all *Zrf1* constructs resulted in a 3-to-5-fold increase in *Zrf1* levels, with the exception of *P{EP}G4964* (Fig 2A). Interestingly, upregulating *Zrf1* in progenitors only led to a moderate increase in ISC proliferation in response to damage but not during homeostasis (Fig 2B). Furthermore, CRISPR/Cas9-mediated transcriptional activation (*egt*^*ts*^* > Cas9.VPR)* of *Zrf1* also led to a damage-specific increase in ISC proliferation (Fig 2C and 2D). These results suggest that Zrf1 is sufficient to increase ISC proliferation only in response to damage.

### *Zrf1* interacts with multiple signaling pathways to regulate ISC proliferation

Bleomycin-induced damage in the gut is known to activate EGFR signaling [15]. Since the loss-of-function and overexpression of *Zrf1* resulted in the most pronounced defects in response to damage, we sought to test if it genetically interacts with the EGFR/Ras/Raf pathway. Ectopic expression of activated EGFR (*TOP4.2*), Ras (*Ras1*^*A*^), Raf (*Raf*^*F149*^) or the human Raf (*hRaf*^*gof*^) in progenitors resulted in ISC overproliferation (Fig 2E, 2G and 2I), which could be rescued by *Zrf1* knockdown (Fig 2F, 2H and 2I), which places it downstream of the EGFR/Ras/Raf pathway. However, *Zrf1* is not restricted to EGFR signaling, since interaction experiments place it downstream of both Notch and Yorkie pathways as well (Fig 2J and 2K). These findings imply a broad function for *Zrf1*, reminiscent of the role of *Myc* in the midgut [11]. A previous study showed that knockdown of *Myc* in ISCs caused stunted proliferation in response to damage, and like *Zrf1*, downregulating *Myc* rescued overproliferation defects caused by various signaling pathways [11]. Thus, we suspected *Zrf1* to be in the same pathway as Myc and found compelling evidence to support this when examining the literature. ChIP-seq of Myc in Kc cells showed that Myc binds to *Zrf1* [16]. In addition, microarrays of larval tissue indicate that *Zrf1* is upregulated following *Myc* overexpression [17]. Finally, co-expression analysis of scRNA-seq data show *Zrf1* as a putative member of the *Myc* regulon [18].

To test whether *Zrf1* is downstream of Myc, we knocked down *Myc* in S2R+ cells which resulted in a decrease in *Zrf1* transcript levels (Fig 2M). Knockdown of *Zrf1* stunted S2R+ proliferation but did not affect the levels of *Myc* (Fig 2L and 2N). Accordingly, *Myc* overexpression increased the levels of *Zrf1*, whereas upregulating *Zrf1* did not affect *Myc* levels (Fig 2O and 2P). In conclusion, our results suggest that *Zrf1* function downstream of *Myc*, integrating multiple signaling pathways to regulate ISC proliferation.

### Zrf1 Protein-Protein interaction network identifies association with RISC

**Fig 3:**
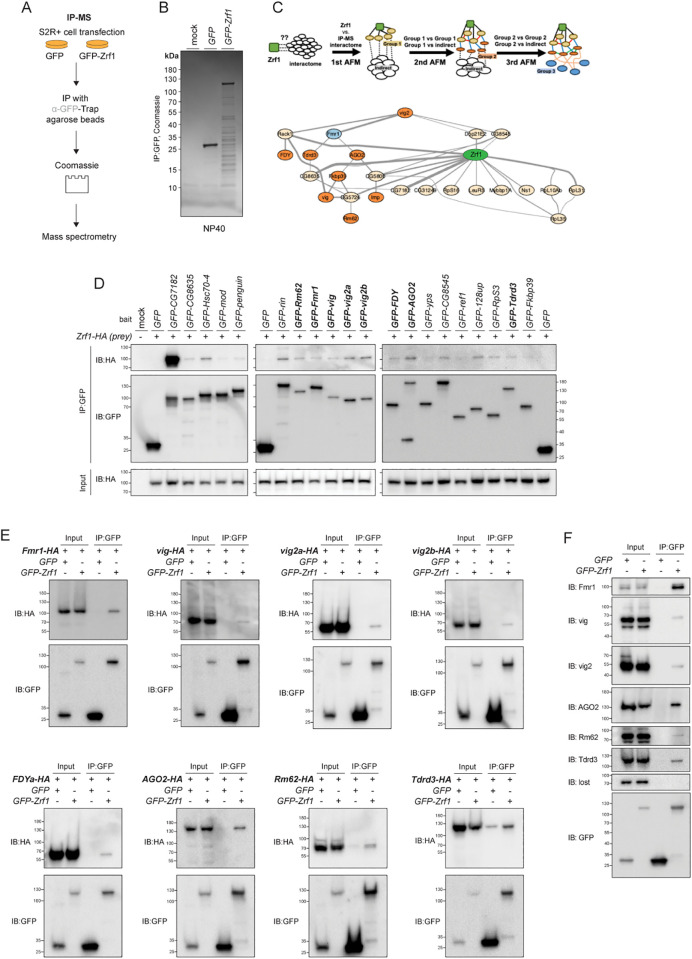
IP-MS-AFM reveals Zrf1 Protein-Protein Interactions (PPI) with components of the RISC components in *Drosophila.* **(A)** Schematic of the experimental workflow for immunoprecipitation-mass spectrometry (IP-MS). S2R+ cells were transfected with constructs expressing either *GFP* or *GFP-Zrf1*. Following cell lysis, GFP-trap agarose beads were used to immunoprecipitate complexes, which were then analyzed via mass spectrometry after Coomassie staining. **(B)** Coomassie-stained gel showing proteins immunoprecipitated with GFP-Zrf1 compared to GFP alone. **(C)** Sequential AlphaFold-Multimer (AFM) screen scheme and protein interaction network generated from the Zrf1 IP-MS interactome analysis. The screening begins with the 1st AFM, predicting interactions between Zrf1 (central node, green) and the entire interactome to identify direct interactors with Zrf1 (Group 1, beige nodes). The 2nd AFM further explores interactions within Group 1 and between Group 1 and the indirect interactome, identifying new interactors (Group 2, orange nodes) and connections within Group 1. Finally, the 3rd AFM involves predictions between Group 2 proteins and additional indirect interactors, forming Group 3 (light blue nodes). Edge thickness represents the Local Interaction Score (LIS), where thicker lines indicate stronger predicted interactions. **(D)** Validation of Zrf1 interactions with candidate proteins identified in IP-MS through co-immunoprecipitation (Co-IP). S2R+ cells were transfected with GFP-tagged candidate proteins and HA-tagged Zrf1. Immunoprecipitation with anti-GFP was followed by immunoblotting with anti-HA to detect Zrf1. Representative interactions with various proteins, including Rm62, Vig, and others, are shown. **(E)** Reciprocal Co-IP with GFP-tagged Zrf1 and HA-tagged components of RISC, such as Fmr1, Vig, Vig2a, and others. **(F)** Immunoblots validating the association of Zrf1 with endogenous RISC components. Proteins including Fmr1, Vig, Vig2, AGO2, Rm62, and Tdrd3 were detected, confirming the interactions of Zrf1 with the RISC complex.

To gain insight into the function of Zrf1, we performed a search for interacting proteins, which we reasoned would identify regulators and localization partners. The Zrf1 protein complex was immunoprecipitated under mild detergent conditions (NP40) using GFP-trap beads from S2R+ cells expressing N-terminally GFP-tagged Zrf1 and subjected to mass spectrometry (IP-MS) (Figs 3A, 2B and S2A-S2C). Because IP-MS captures both direct binders and indirect co-complex members, we applied the AFM-LIS (AlphaFold-Multimer-Local Interaction Score) analysis pipeline in three iterative rounds to predict high-confidence direct interactors (Fig 3C) [19]. In Round 1, Zrf1 was modeled against each MS candidate, and only models exceeding a Local Interaction Score (LIS) of ≥ 0.203 and a Local Interaction Area (LIA) of ≥ 3432 were retained as primary interactors. In Round 2, these primary hits were screened against the subset of MS candidates that had failed in Round 1 to uncover bridging (“secondary”) interactions. In Round 3, the remaining MS candidates were modeled against the consolidated core set to reveal tertiary contacts. Ultimately, a Zrf1 PPI network comprising approximately 40 direct PPIs was built. Details for whole prediction results and predicted aligned error maps for all positive PPI predictions are shown in  S3 Fig. Network analysis revealed associations with ribosomal proteins and components of the RNA-induced silencing complex (RISC) among other proteins (Fig 3C). Consistent with this, Zrf1 IP-MS showed >2-fold spectral counts for Rm62, Fmr1, Vig, Vig2, AGO2 and Tdrd3 in comparison to controls. To test whether these proteins interact with Zrf1, we conducted co-immunoprecipitation (Co-IP) experiments with S2R+ cells transfected with C-terminally 3xHA-tagged Zrf1 and N-terminal-GFP-tagged binding partners (Fig 3D). In total, Zrf1-3xHA immunoprecipitated with 17 out of 19 GFP-tagged binding partners. This included CG7182, CG8635, Hsc70–4, mod, penguin, rin, Rm62, Vig, two isoforms of Vig2, FDY (a Y-chromosome duplication of *vig2*), CG8545, 128up and Tdrd3. Reciprocal Co-IP found that C-terminal-3xHA-tagged Rm62, Fmr1, Vig, Vig2a, Vig2b, AGO2, FDY and Tdrd3 precipitated with GFP-Zrf1 (Figs 3E and S4A-S4D). Antibodies against these proteins showed that endogenous levels also immunoprecipitate with Zrf1, indicating a robust physical association between Zrf1 and RISC components (Figs S4E and 3F).

To determine if the physical interactions between Zrf1 and RISC was conserved in humans, we conducted IP-MS on N- and C- terminally epitope-tagged ZRF1 in HEK293T cells (under mild detergent conditions) (S5A-S5C Fig). The MS spectrum counts of both tagged versions of ZRF1 were enriched for SERBP1 (ortholog of Vig, Vig2 & FDY), human orthologs of Rm62 (DDX17, DDX21 and DDX5) or Fmr1 (FXR1, FMRP and FXR2), but not for TDRD3, HABP4 or members of the AGO-family (S5D Fig). Nonetheless, we conducted Co-IP experiments to determine if the PPIs of Zrf1 are conserved. C-terminal-epitope-tagged SERBP1, HABP4, DDX17, DDX21, or DDX5 all precipitated with Flag-ZRF1 (S5F Fig top). ZRF1-GFP precipitated with N-terminal-Flag-tagged AGO1 (S5F Fig bottom). Reciprocal Co-IP confirmed the ZRF1-SERBP1 and ZRF1-HABP4 interactions. Additionally, IP with a ZRF1 antibody successfully detected endogenous FXR1, FMRP and FXR2 (S5E Fig).

### Components of RISC genetically interact with *Zrf1* to regulate ISC proliferation

**Fig 4:**
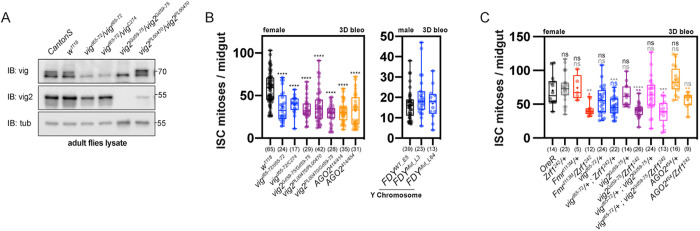
Genetic interaction between *Zrf1* and RISC components regulates ISC proliferation during midgut damage. **(A)** Western blot analysis of adult fly lysates showing protein levels of Vig and Vig2 in various genetic backgrounds. Samples include wild-type controls (*Canton-S, w*^*1118*^), and mutant alleles for *vig* (*vig*^*d65-72*^*, vig*^*d63-73*^*, vig*^*C274*^), *vig2* (*vig2*^*Gd59-75*^*, vig2*^*PL00470*^), and a double mutant (*vig2*^*PL00470*^*/vig2*^*Gd59-75*^). Tubulin (tub) was used as a loading control. The blots confirm the effective reduction of Vig and Vig2 in the mutant backgrounds. **(B)** ISC mitoses per midgut after 3 days of bleomycin (bleo) treatment in various female and male genotypes. Mutants for *vig* and *vig2* exhibit a reduction in ISC proliferation compared to controls (*w*^*1118*^). Mutants for *AGO2* (*AGO2*^*414/414*^ and *AGO2*^*414/454*^) also show decreased proliferation. See also S1 Data Table **U. (C)** ISC mitoses per midgut after 3 days of bleomycin treatment in double heterozygous animals. Females carrying *Zrf1*^*242*^*/ +* combined with heterozygous mutant alleles for RISC components (*Fmr1*^*d113M*^*, vig*^*d65-72*^*, vig*^*d63-73*^*, vig2*^*Gd59-75*^, and *AGO2*^*454*^) are compared to individual heterozygotes. Significant reductions in ISC proliferation are observed in certain double heterozygotes, suggesting a synergistic interaction between *Zrf1* and these RISC components. Error bars represent the interquartile range; n = number of midguts analyzed for each genotype. See also S1 Data Table **V.** Box plots show median, quartiles, and range. Statistical significance determined by Student’s t-test; ns = not significant, **p < 0.01, ****p < 0.0001.

We hypothesized that perturbing components of RISC would mimic the effects of *Zrf1* knockdown. Utilizing CRISPR, we generated mutants for *vig* and *vig2*, and assessed ISC proliferation during gut damage (Fig 4A). Homozygous and transheterozygous mutants of *vig*, *vig2*, and *AGO2* exhibited reduced ISC proliferation in response to bleomycin, while FDY mutants showed no defects (Fig 4B). Additionally, double heterozygous animals — *Zrf1*^*242*^/+ with either *fmr1*(*fmr1*^*d113M*^*/Zrf1*^*242*^), *vig2* (*vig2*^*Gd59-75*^/*Zrf1*^*242*^), or *AGO2* (*AGO2/ + ;Zrf1*^*242*^*/+*) —displayed a synergistic decrease in ISC proliferation compared to individual heterozygotes (Fig 4C). These results collectively suggest a genetic interaction between *Zrf1* and the RISC components (*Fmr1*, *vig2*, and *AGO2*) pivotal for regulating ISC proliferation during damage recovery.

### *Zrf1* is not involved in RNAi-mediated post-transcriptional silencing

**Fig 5:**
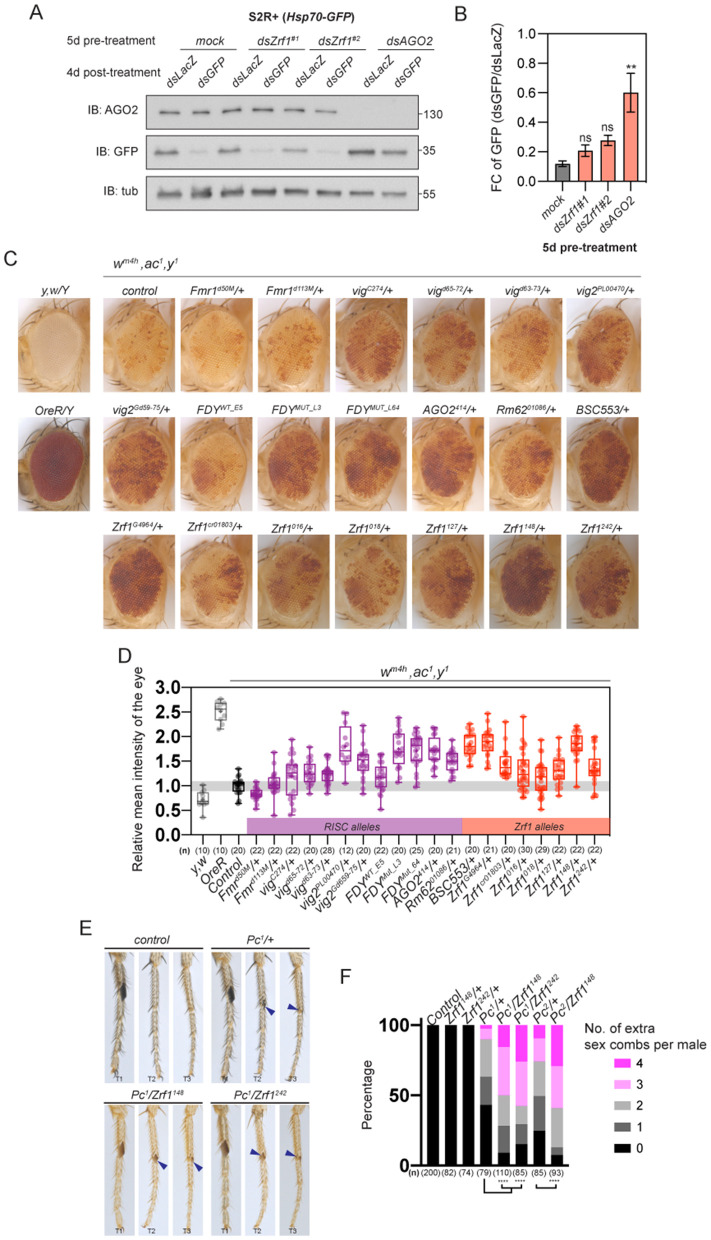
*Zrf1* mutants exhibit deregulation of chromatin-associated defects. **(A)** Western blot analysis of S2R+ cells pre-treated for 5 days with various dsRNAs (*dsLacZ, dsZrf1, dsAGO2*). Cells were subsequently treated with *dsGFP* to assess RNAi-mediated silencing. Immunoblots show levels of AGO2, GFP, and tubulin (loading control). **(B)** Quantification of GFP levels from **(A)**, normalized to tubulin. Knockdown of *Zrf1* did not significantly change GFP silencing efficiency, supporting that Zrf1 is not involved in RNAi-mediated post-transcriptional silencing. See also S1 Data Table **W. (C)** Representative images of fly eyes of *w*^*m4h*^*, ac*^*1*^*/ Y* males crossed with various RISC and *Zrf1* mutants. **(D)** Quantitative analysis of relative mean pigmentation intensity from **(C)**. Box plots compare pigmentation across RISC and *Zrf1* mutant backgrounds, normalized to control (*w*^*m4h*^*, ac*^*1*^*/Y*). Reduced pigmentation corresponds to enhanced suppression of PEV, while increased pigmentation indicates loss of PEV suppression. See also S1 Data Table **X. (E)** Extra sex comb phenotypes in *Pc*^*1*^ and double heterozygous *Pc*^*1*^*; Zrf1* mutant males. Images show T2 and T3 legs of control (*Pc*^*1*^*/+*) and double heterozygous (*Pc*^*1*^*/Zrf1*^*148*^*, Pc*^*1*^*/Zrf1*^*127*^) flies. Blue arrows point to the presence of ectopic sex combs, indicating synergistic enhancement of *Pc*^*1*^ phenotypes by *Zrf1* mutants. **(F)** Percentage distribution of extra sex combs in *Pc*^*1*^ and double heterozygous *Pc*^*1*^*; Zrf1* mutant males. Bar graph displays the proportion of males with 0, 1, 2, 3, or 4 extra sex combs per male across genotypes, with *Zrf1* mutants showing an increased frequency of ectopic sex combs. See also S1 Data Table **Y.** Box plots display median, quartiles, and range; statistical significance determined by Student’s t-test; ns = not significant, **p < 0.01, ***p < 0.001, ****p < 0.0001.

In light of the established roles of *AGO2* and *Rm62* in RNAi-mediated post-transcriptional silencing [20,21] and the physical association between Zrf1 and RISC, we posited that Zrf1 might play a role in the RNAi pathway. Thus, we assessed the knockdown efficiency of *dsGFP* in S2R+ cells expressing *GFP* following pre-treatment with various dsRNA. Initially, cells were exposed to either *dsLacZ* (control), *dsAGO2*, or *dsZrf1*. After 3–5 days, the same cells were subjected to *GFP* dsRNA treatment or left untreated. The effectiveness of RNAi-mediated silencing was assayed by measuring GFP levels normalized to tubulin. As anticipated, RNAi-mediated *GFP* knockdown was compromised in cells pre-treated with *dsAGO2* compared to the control. Intriguingly, knockdown of *Zrf1* using two independent dsRNAs did not impact *GFP* knockdown (Fig 5A and 5B). Our findings indicate that *Zrf1* does not participate in RNAi-mediated post-transcriptional silencing.

### *Zrf1* mutants dominantly suppress position-effect variegation and enhance polycomb defects

Previous studies have revealed that RISC components have non canonical roles in modifying heterochromatin gene silencing [22,23]. To investigate whether Zrf1 shares similar functions, we used the positive-effect variegation (PEV) reporter *In(1)w*^*m4h*^, where the *white* (*w*) gene is transposed close to pericentromeric heterochromatin on the X-chromosome by a large chromosome inversion [24]. PEV results in suppressed *w*^*+*^ expression in *w*^*m4h*^*/Y* males, leading to mottled eye pigmentation (Fig 5C). Conventionally, enhancers and suppressors of PEV have been associated with genes that regulate chromatin condensing and de-condensing, respectively. Confirming earlier studies [25,26], *AGO2* (*AGO2*^*414*^*/+*) and *Rm62* (*Rm62*^*01086*^*/+*) mutants suppressed *w*^*m4h*^ PEV, while *Fmr1* (*Fmr1*^*d50M*^*/ +* & *Fmr1*^*d113M*^*/+*) mutations did not. In addition, *vig* alleles *(vig*^*d65-72*^*/ +* & *vig*^*d63-73*^*/+* but not *vig*^*C274*^*/+*) moderately suppressed PEV, whereas *vig2* alleles (*vig2*^*PL00470*^ & *vig2*^*Gd59-75*^) exhibited strong suppression. Finally, *FDY* mutations (*FDY*^*MUT_L3*^ & *FDY*^*MUT_L64*^) also dominantly suppressed PEV when compared to the control (*FDY*^*WT_E5*^) (Fig 5C and 5D).

Similar to the components of RISC, heterozygous alleles of *Zrf1* dominantly suppressed *w*^*m4h*^ PEV. This encompassed a deficiency that covers *Zrf1* (*BSC553*), a P-element insertion with a weak mini-white (*Zrf1*^*G4964*^) and five indel mutants (*Zrf1*^*016*^*, Zrf1*^*018*^*, Zrf1*^*127*^*, Zrf1*^*148*^ & *Zrf1*^*242*^) generated using CRISPR/Cas9 (Figs 5C, 5D and S6A). Collectively, our findings unveil Zrf1 and RISC components (except Fmr1) as dominant suppressors of PEV, suggesting a broad role in chromatin condensation.

Notably, Polycomb group proteins are also known to regulate transcriptional repression of target genes through chromatin modifications. A previous study highlighted the ability of ZRF1 to bind to H2Aub1 and displace PRC1, activating previously repressed genes [10]. Another study also showed that plant ZRF1 possesses both PRC1-related and PRC1-unrelated functions [27]. Given this context, we hypothesized that Zrf1 might interact with Polycomb (Pc), influencing chromatin structure and gene expression. Using *Pc*^*1*^ animals, a weak amorphic allele of *Pc*, we tested for genetic interactions between *Zrf1* and *Pc.* In wildtype flies, sex combs are only found at the distal end of the male foreleg (T1) (Figs 5E and S6B). However, heterozygous *Pc*^*1*^ males have varying degrees of extra sex combs in the second (T2) and third (T3) legs. *Zrf1* heterozygous mutant males (*Zrf1*^*148*^*/ + , Zrf1*^*242*^*/+*) are indistinguishable from control animals. Interestingly, double heterozygous animals of *Zrf1* and *Pc* alleles (*Zrf1*^*148*^*/Pc*^*1*^*, Zrf1*^*242*^*/Pc*^*1*^) synergistically enhanced the sex comb defect observed in *Pc*^*1*^ heterozygous animals alone (Figs 5E, 5F and S6B), suggesting that Zrf1 enhances the de-repressed genes regulated by Pc.

## Discussion

Our study has uncovered a critical role for Zrf1 in regulating intestinal stem cell (ISC) proliferation in the *Drosophila* midgut. By leveraging a combination of genetic tools, IP-MS, and AlphaFold-Multimer predictions, we provide comprehensive insights into the multifaceted functions of Zrf1.

### Zrf1 as a key regulator of ISC proliferation

Although *Zrf1* orthologs share some conserved roles across species, their functions have diverged considerably. In mammals, ZRF1 displaces Polycomb complexes to activate developmental genes critical for cell fate transition [10]. Similarly, in plants, *Zrf1* orthologs promote the activation of genes associated with flowering and growth [28,27]. By contrast, the yeast ortholog Zuotin operates as a molecular chaperone within the ribosome-associated complex, assisting nascent polypeptide folding and maintaining proteostasis [29]. The functional differences highlight the unique value of *Drosophila* as a model to uncover aspects of Zrf1 biology that may be obscured in other organisms.

Our results establish that Zrf1 is essential for ISC proliferation during both homeostasis and regeneration. Our RNAi screen identified Zrf1 as a critical factor, with its knockdown leading to a significant reduction in ISC proliferation. This phenotype was not rescued by blocking apoptosis, indicating a specific requirement for Zrf1 in ISC proliferation rather than cell survival. Interestingly, *Zrf1* overexpression promoted ISC proliferation only in response to damage, suggesting that its role is tightly regulated by cellular context and environmental cues.

The integration of Zrf1 into multiple signaling pathways, including the EGFR/Ras/Raf, Notch, and Yorkie pathways, underscores its broad regulatory capacity. Our interaction experiments position Zrf1 downstream of these pathways, akin to the role of Myc in the midgut. This finding aligns with previous studies showing that Myc and Zrf1 share common regulatory networks [11], further supported by ChIP-seq and microarray data [17,16]. Altogether, our results suggest that Myc likely regulates the direct transcription of Zrf1.

### Zrf1 protein-protein interaction network and association with RISC

The construction of a Zrf1 protein-protein interaction network using AlphaFold-Multimer and Local Interaction Score (LIS) analysis revealed significant associations with components of the RNA-induced silencing complex (RISC) [19]. Co-IP experiments confirmed these interactions, highlighting the potential involvement of Zrf1 in RNA processing and silencing mechanisms. However, our functional assays demonstrated that Zrf1 does not participate in RNAi-mediated post-transcriptional silencing, redirecting our focus toward its chromatin-related functions.

### Zrf1 as an epigenetic factor

Our findings that *Zrf1* mutants dominantly suppress position-effect variegation (PEV) and enhance Polycomb (Pc) defects provide compelling evidence for a role of Zrf1 in epigenetic regulation. The synergistic interaction between *Zrf1* and *Pc*^*1*^ alleles, resulting in enhanced sex comb phenotypes, suggests that Zrf1 modulates chromatin structure and gene expression in concert with Pc group proteins. This is consistent with previous reports indicating the ability of Zrf1 to displace Pc complexes from chromatin to activate gene expression [10]. Interestingly, other chromatin regulators such as Piwi have also been implicated in maintaining heterochromatin organization and repressing transposable elements in ISCs [30,31]. Although we did not detect a direct interaction between Zrf1 and Piwi in our study, both factors may act through parallel pathways to safeguard chromatin integrity in ISCs.

### Implications for cancer and therapeutic potential

The role of Zrf1 in regulating ISC proliferation and chromatin dynamics suggests potential implications for cancer biology. Given that Zrf1 can function as both a tumor suppressor and an oncogene depending on the context, understanding its regulatory networks could provide new targets for cancer therapy [32,33]. For example, in the context of gastric carcinoma, where *Zrf1* is overexpressed [34], targeting its interaction with Pc and RISC components could offer novel therapeutic strategies.

### Future directions

Future research should aim to elucidate the precise molecular mechanisms underlying Zrf1’s interactions with Pc and RISC components. Additionally, investigating Zrf1’s role in other tissues and cancer types will be crucial for translating these findings into clinical applications. Understanding how Zrf1 integrates multiple signaling pathways to regulate stem cell behavior and chromatin dynamics will provide valuable insights into stem cell biology and open new avenues for therapeutic development.

In conclusion, our study highlights the critical role of Zrf1 in ISC proliferation and chromatin regulation, with significant implications for stem cell biology and cancer therapy. By unraveling the complex networks involving Zrf1, this study paves the way for future research and potential clinical applications targeting Zrf1 and its associated pathways.

## Methods

### RNAi screening

We used *esg-Gal4, UAS-GFP, tub-Gal80*^*ts*^ (*egt*^*ts*^) to knockdown genes in adult ISCs/EBs and quantified ISC proliferation by counting the number of mitotic (pH3-positive) cells per midgut. *UAS-RNAi* lines were obtained from Bloomington (BDSC), Vienna (VDRC) and NIG/Kyoto *Drosophila* stock centers. *UAS-RNAi* stocks that are homozygous lethal on second chromosomes were placed over *CyO, twi-Gal4, UAS-2EGFP* balancers. Virgin *egt*^*ts*^ females were crossed to *UAS-RNAi* males and reared at 18°C (restrictive temperature). After eclosion, *egt*^*ts*^ + *UAS-RNAi* females were kept at 18°C for 3 days and shifted to 29°C (permissive temperature) for 10 days before their whole midguts were dissected. Flies were transferred to fresh vials every 2–3 days. Mated females were used in all experiments and reared with male flies at the ratio of 2:1. Whole midguts were analyzed for pH3 positive counts.

### Infection and compound feeding

The OD_600_ of an overnight suspension of *Ecc15* was determined by measuring two diluted (1:10 & 1:100) aliquots of the culture. After centrifuging the culture at 3000g for 20min, the pellet was resuspended in 5% sucrose with a volume to a final OD_600_ of 100. Paper towels cut out to the shape of the vial base were soaked with 500uL of the concentrated *Ecc15* solution. For oral infection, flies were starved for 2h at 29°C in an empty vial before transferring to vials containing *Ecc15*.

### Immunohistochemistry and imaging

Whole midguts were dissected in PBS and fixed in 4% PFA in PBS at room temperature for 30 minutes. Fixed guts were washed in 0.1% Triton X-100 in PBS (PBST) once then blocked with a blocking buffer (0.1% Triton, 5%NDS in PBS) for 30 minutes at RT. Primary antibodies were incubated overnight at 4°C in the blocking buffer. Guts were washed 3x in a blocking buffer and incubated with secondary antibodies overnight at 4°C along with DAPI (1:1000 of 1mg/ml stock). After antibody staining, guts were washed 3x in PBST and mounted in Vectashield antifade mounting medium (Vector Laboratories H-1200). Tape was used as a spacer to prevent coverslips from crushing the guts. Antibody dilutions used were as follows: rabbit anti-pH3 (1:1000, Millipore 06–570), mouse anti-Dachshund (1:20, DSHB AB_579773), chicken anti-GFP (1:1000, Abcam ab13970), donkey anti-rabbit 565 (1:2000, Molecular Probes A31572), goat anti-mouse 633 (1:2000, Thermo Scientific A-21240) and goat anti-chicken 488 (1:2000, Thermo Fisher Scientific A-11039). Guts were imaged on a spinning-disk confocal system, consisting of a Nikon Ti2 inverted microscope equipped with a W1 spinning disk head (50um pinhole, Yokogawa Corporation of America) and a Zyla 4.2 Plus sCMOS monochrome camera (Andor).

### RNA extraction and quantitative real-time PCR

10-15 midguts were dissected in 1xPBS and homogenized in 300uL of TRIzol (ThermoFischer, 15596–026) using RNase-free pestles. RNA was extracted using Zymo Direct-zol RNA MicroPrep kit (R2050) and subsequently DNase-treated using Turbo DNA free (AM1907). 400–450ng of the resulting RNA was reverse transcribed using Bio-Rad iScript Select cDNA synthesis kit (708896) and Sybr green (1708880) based qRT-PCR was performed to determine the levels of gene expression. qRT-PCR primers were designed using FlyPrimerBank.^30^ Primers that were not from the literature were tested for their efficiency by running qRT-PCR on serial dilutions of pooled cDNA. Only primers with efficiencies of greater than 85% were selected for further use. The primer pairs tested are as follows: *Zrf1* (PP12004: 81.2%, PP24706: 80.3%, PP36474: 96.0%), *RpL32* (PD41810: 99.6%), *myc* (PP17271: 75.9%, PP29594: 102.1%, PP4159: 94.9%).

### Cell culture and RNA interference

*Drosophila* S2R+ cells were cultured at 25°C in Schneider’s medium with 10% fetal bovine serum (FBS) and streptomycin. HEK293T cells were cultured at 37°C in DMEM (Invitrogen 10-017-CV) supplemented with 10% FBS and streptomycin. For RNAi experiments, DNA templates for dsRNA were obtained from the *Drosophila* RNAi screening center (DRSC). Amplicons used in this study were: *Zrf1*(#11620), *Myc* (#23360, #37533), *Fmr1* (#26781), *vig* (#03631), *vig2* (#14402), *AGO2* (#10847), *Rm62* (#25109), *Tdrd3* (#10016) and *LacZ* dsRNA. A second dsRNA *Zrf1* template was generated by amplifying *Zrf1* cDNA with primers containing overhanging T7 sequences (JL278 and JL279). DNA templates were amplified with T7 primers using Phusion polymerase. PCR products were *in vitro* transcribed (IVT) using MEGAscript T7 Transcription Kit (ThermoFisher, AMB1334-5). S2R+ were treated with dsRNAs by the “bathing” method (https://fgr.hms.harvard.edu/drsc-cellrnai).

JL278: TAATACGACTCACTATAGGGTCTGGAGCAATGAGAACGTG

JL279: TAATACGACTCACTATAGGGGGTTGACTGATTGTTCGCCT

### Cell viability

1x10^5^ cells were plated into 96-well plates. Treated with 500ng of dsRNAs in a total volume of 80ul. 10ul was used and counted for cell number with a haemocytometer.

### Plasmids. Molecular cloning – *Drosophila* constructs

***pEntr-GFP-Stop***: The *pEntr-dTOPO* backbone was amplified from *pEntr-Akt* (Gift from P. Saavedra) with the primers pEntr_F & pEntr_R. GFP was amplified from the plasmid *pAW-vhh05-GFP* (gift from J. Xu) with JL201 and JL202. The two fragments were joined by Gibson assembly (NEB, E2611S). ***pEntr-GFP-Zrf1-Stop****:* The backbone of *pEntr-GFP-stop* was PCR amplified with the primers pEntr_F & JL203. The *Zrf1* coding sequence was amplified from the vector LD23875 with JL204 & JL205. The linker (GGGSGSG) was introduced between the GFP and *Zrf1* by overhanging sequences on the primers. Amplicons were joined by Gibson assembly. ***pEntr-Zrf1-NoStop:*** The *pEntr-dTOPO* backbone was amplified from *pEntr-GFP-stop* with the pEntr_F & pEntr_R. The *Zrf1*coding sequence was amplified from the plasmid LD23875 with JL171 & JL172 (primers that omitted the stop codon). Amplicons were joined by Gibson assembly. ***pEntr-AGO2-RB-NoStop***: The AGO2 coding sequence was amplified from the plasmid *pAFW-AGO2* (Addgene #50554) with the primers JL267 & JL268. The fragment was inserted into the *pEntr* backbone via Gibson assembly. ***pEntr-FDY-NoStop***: Two gBlock (IDT) dsDNA fragments (gJL1 & gJL2) encoding the entire *FDY* coding region (Accession KR781487) were joined with the *pEntr* backbone using Gibson assembly. ***pEntr-Tdrd3-NoStop***: The *Tdrd3* coding sequence was amplified from the plasmid RE1471 with JL293 & 294. ***Final vectors:*** *pEntr223* vectors containing full-length cDNAs of *dMyc*, *Hspa14 (CG7182)*, *Zc3h15* (*CG8635*), *Hsc70–4*, *mod*, *peng*, *Fkbp39*, *Rm62*, *rin*, *Fmr1*, *vig*, *vig2, Ref1, RpS3, 128up, CG8584* and *yps* were from the FlyBi ORFeome Collection. The *pEntr-mCherry-NoStop* was a gift from B. Xia. All fly *pEntr*vectors containing cDNAs were transferred into the *Drosophila* gateway vectors *pAW, pAWH, pAWM* and *pAGW* (Carnegie Science/Murphy lab) using LR clonase II enzyme mix (Invitrogen, 11791–020) (see table for full list of vectors*).*

JL171: CCGCGGCCGCCCCCTTCACCATGACGAGCGGTACGGTAGC

JL172: GGGTCGGCGCGCCCACCCTTTTTGACCGCCGCCTGTG

JL201: CCGCGGCCGCCCCCTTCACCATGGTGAGCAAGGGCGAG

JL202: GGGTCGGCGCGCCCACCCTTTCACTTGTACAGCTCGTCCATGCC

JL203: GCCGGAGCCGCTGCCACCTCCCTTGTACAGCTCGTCCATGC

JL204: GGAGGTGGCAGCGGCTCCGGCATGACGAGCGGTACGGTAGC

JL205: GGGTCGGCGCGCCCACCCTTTCATTTGACCGCCGCCTG

JL267: CCGCGGCCGCCCCCTTCACCATGGGAAAAAAAGATAAGAACAAGAAAGGAG

JL268: GGGTCGGCGCGCCCACCCTTGACAAAGTACATGGGGTTTTTCTTCA

JL293: CCGCGGCCGCCCCCTTCACCATGGAATTAGGCAAGAAACTACG

JL294: GGGTCGGCGCGCCCACCCTTATGCTCTCGTTTGTGGG

### Molecular cloning – Human constructs

***pEntr-ZRF1-NoStop***: The human *ZRF1* coding sequence was amplified from the plasmid *pcDNA3.1-ZRF1* (GenScript, OHu13274) with JL186 & JL188. ***pEntr-SERBP1-NoStop:*** The human SERBP1 coding sequence was amplified from the plasmid UAS-SERBP1 (DGRC, hGUHO03824) with JL313 & JL314. ***Final vectors:*** *pDonr221-HABP4-NoStop* (DNASU HsCD00745639), *pEntr-mCherry-NoStop* and other *pEntr* vectors containing cDNAs were transferred into the mammalian gateway vectors, *pHAGE-N-FLAG-HA* (*pHFH;* gift from R. Viswanatha) or pCSF107mT-GATEWAY-3’LAPtag (*pCSF-GFP*, Addgene #67618, gift from S. Entwisle) using LR clonase II enzyme mix. Other plasmids containing epitope-tagged cDNAs were used and are listed as follows*: *pDEST-myc-DDX17** (Addgene #19876).

JL186: CCGCGGCCGCCCCCTTCACCATGCTGCTTCTGCCAAGCG

JL188: GGGTCGGCGCGCCCACCCTTTCATTTCTTGGCTCTACTTGCATTCAGCA

JL313: CGCGGCCGCCCCCTTCACCATGCCTGGGCACTTACAGGAAG

JL314: GGGTCGGCGCGCCCACCCTTAGCCAGAGCTGGGAATGCC

### Immunoprecipitation and immunoblotting

Immunoprecipitation was performed as previously described.^31^ S2R+ or HEK293T cells were split 2 days before transfection. 2ug of DNA was transfected into 20 x 10^6^ of cells in a 100mm dish with Effectene transfection reagent (Qiagen 301427) following the manufacturer’s protocol. After 3 days, cells were lysed with IP-lysis buffer (Pierce 87787) containing 2X protease inhibitor cocktail (ThermoFisher, 78430) and 2X PhosSTOP (Sigma-Aldrich 4906837001). Lysate was incubated with 25ul of Chromotek-GFP-trap beads (Bulldog Biotechnology gta-20) or anti-Flag agarose (Sigma A2220) and subject to end-over-end agitation for 2hrs at 4°Cs. Beads were washed 3–4 times with 500ul of lysis buffer. Beads were eluted in 25ul of sample buffer (Pierce 39001 with 10% 2-Mercaptoethanol), boiled for 5min and sedimented by centrifugation at 2500x g for 5min.

For immunoblotting, samples were run on 4%-20% polyacrylamide gel (Bio-Rad 4561096) and transferred to an Immobilon-P polyvinylidene fluoride (PVDF) 0.45um membrane (Millipore IPVH00010). The membrane was blocked with 5% skim milk in TBST (TBS with 0.05% Tween-20) at room temperature for 1h and probed with primary antibody in 5% skim milk overnight at 4°C. Membrane was probed with HRP-conjugated secondary antibody at room temperature for 1h and signal detected by enhanced chemiluminescence (ECL;Amersham RPN2209; Pierce 34095). Concentrations used for primary antibodies were as follows: chicken anti-GFP (1:10000, aveslabs GFP-1020), use anti-HA.11 (1:1000, BioLegend 901514), mouse anti-Fmr1 (1:100, DSHB 5A11), rabbit anti-Vig (1:30000, gift from N. Riddle) & rabbit-Vig2 (1:10000, gift from N. Riddle), mouse anti-dAGO2 (1:400, gift from H. Siomi), rat anti-Rm62 (1:2000, gift from E. Lei), rabbit anti-Tdrd3 (1:2000, gift from W. Wang), mouse anti-lost (1:100, DSHB), mouse anti-Tubulin (1:1000, Sigma T5168).

### Measuring eye pigmentation

*w*^*m4h*^*, y*^*1*^*, ac*^*1*^ females were crossed with different alleles and reared at 25°C. In the progeny, the left eye of 2-day-old adult male flies were imaged and used for measuring eye pigmentation. Images were separated into the RBG channels, and the mean grey intensity of the eye was measured under the red channel. This value was subtracted from the background mean grey intensity and compared between genotypes as a fold-change of the *w*^*m4h*^*, y*^*1*^*, ac*^*1*^ control.

### Generating fly mutants with CRISPR

***vig* mutants:** *y; P{TKO.GS04822}*attP40 (BL81492) was crossed with *y,w; nos-Cas9/CyO*. *y,w; nos-Cas9/ P{TKO.GS04822}*attP40 males were crossed with *y,w; Gla/CyO* females. Resulting offsprings were outcrossed individually to establish stocks. A ~ 500 bp region covering the seed sequence was amplified with JL317 & JL318 and subcloned into pJET1.2/blunt vector for sequencing. The selected mutants, *vig*^*d65-72*^ and *vig*^*d63-73*^ have predicted premature stop codons that result in respectively truncated proteins of 59aa and 58aa.

Zrf1 mutants: *y,w;P{HD_CFD0021}attP40/CyO-GFP (v341280)* was crossed with *y,w; nos-Cas9/CyO.* Male *y,w; nos-Cas9/attP40* was crossed with *y,w; IF/CyO*. Resulting offspring were outcrossed individually to establish stocks. Established stocks were crossed with a deficiency stock. Stocks resulting in non-complementation were deemed mutants.

JL317: ATCGAATCTCACAGGGTGTGAGAG

JL318: GTTTGTTGTCGGCCGATGGA

***vig2* mutants:** *y; P{TKO.GS04324}attP40* (BL84243) was crossed with *y,w; nos-Cas9/CyO*. *y,w; nos-Cas9/ P{TKO.GS04324}attP40* males were crossed with *y,w; TM3/TM6b* females. Resulting offsprings were outcrossed individually to establish stocks. A ~ 600 bp region covering the seed sequence was amplified with JL315 & JL316 and subcloned into pJET1.2/blunt vector for sequencing. The selected mutants, *vig2*^*d53-72*^, *vig2*^*d57-66*^ and *vig2*^*G.d59-75*^, have predicted premature stop codons that result in respectively truncated proteins of 61aa, 26aa and 25aa.

JL315: GCCATTGGGACCAAAGTAACTCTCAAA

JL316: TTACAGGGCAGCATTTTCCACG

### Sampling and statistics

Experimental data was always collected alongside a control group. Most if not all experiments were conducted once with at least a sample size of three. All statistics were collected in the traditional frequentist fashion. The Shapiro-Wilk test was used to test normality. For experiments comparing only two groups, two-tailed unpaired t-test were used. Ordinary one-way ANOVA for parametric multiple comparison and the non-parametric Kruskal-Wallis test was used for comparing two or more groups. Subsequent Dunn’s multiple comparison test was used to compare each group with the control.

## Supporting information

S1 Fig Midgut progenitor RNAi screen dientifies *Zrf1.* (A) ISC mitoses per midgut from an RNAi screen targeting 119 DNA-binding proteins (*UAS-DBP-RNAi*) driven by *egt*^*ts*^* > GFP*. Each bar represents ISC mitoses for a specific RNAi line after 10 days at the permissive temperature. Red bars indicate lines with significantly reduced ISC mitoses compared to control (Luc-i). The gray bar is the Control (EGT/Luc-i). *Zrf1* RNAi (*Zrf1-i*) is among the top hits causing a strong reduction in ISC proliferation. (B–C) Confocal images showing GFP + ISC/EB progenitor cells after 10 days of RNAi induction in *egt*^*ts*^*F/O* flies during homeostasis. (B) Control (*Luc-i*) showing normal ISC proliferation, and (C) *Zrf1* knockdown (*Zrf1-i*) showing a marked reduction in GFP+ progenitor cells. Green: GFP; blue: DAPI. Scale bar: 50 µm. (D) Quantification of ISC mitoses per midgut from *egt*^*ts*^*F/O* flies after 10 days of RNAi induction during homeostasis or bleomycin (bleo) treatment. (E) ISC mitoses per midgut from *DI*^*ts*^ flies where *Zrf1* RNAi was specifically driven in ISCs. ISC proliferation is markedly reduced upon *Zrf1* knockdown compared to control (*Luc-i*), both during homeostasis and after bleomycin treatment. Bars represent mean ± SEM; n = number of midguts analyzed. Statistical significance determined by Student’s t-test; ***p < 0.001, ****p < 0.0001.*.
(TIF)

S2 Fig Validation of Zrf1 interaction network by IP-MS and network analysis. (A) Western blot analysis of immunoprecipitated (IP) samples from S2R+ cells expressing GFP or GFP-Zrf1 across three biological replicates (Rep 1, Rep 2, Rep 3). IP was performed using GFP-trap beads, and the presence of GFP-tagged proteins was confirmed by immunoblotting (IB) for GFP. Input samples show consistent expression levels across all replicates. (B) Coomassie-stained gels of IP samples from S2R+ cells expressing GFP or GFP-Zrf1. Three biological replicates (Rep 1, Rep 2, Rep 3) are shown, demonstrating consistent patterns of protein bands enriched in the GFP-Zrf1 lanes, confirming the reproducibility of Zrf1-associated protein complexes. Samples were prepared under mild detergent (NP40) conditions. (C) Additional Coomassie-stained gels highlighting the distinct protein bands observed in GFP-Zrf1 IP samples across the three replicates (Rep 1, Rep 2, Rep 3). This suggests specific enrichment of proteins that associate with Zrf1. (D) Network diagram showing the Zrf1 PPI network derived from IP-MS data. Nodes represent proteins, with colors indicating the SAINT score (green: high, yellow: low). Edges represent interactions, with line colors indicating MIST rank confidence (red: high, pink: moderate, gray: low). The network reveals associations between Zrf1 and multiple ribosomal proteins, RNA-binding proteins, and components of the RNA-induced silencing complex (RISC), highlighting the diverse interactome of Zrf1. IP-MS data validation was performed across three independent biological replicates, confirming reproducible interaction profiles. The network was constructed using SAINT and MIST scoring to assess interaction confidence.
(TIF)

S3 Fig AlphaFold-Multimer predicted aligned error (PAE) maps for Zrf1 interactome predictions. PAE maps of positive PPIs using AlphaFold-Multimer (AFM) screens to predict direct interactions between Zrf1 (CG10565) and candidate interactors identified from IP-MS data. Each PAE map represents the residue-residue alignment confidence between Zrf1 and a candidate interactor. In these PAE maps, blue regions indicate low predicted alignment error, suggesting high confidence in residue-residue alignment (potential interaction), while red regions denote high alignment error, indicating lower confidence. Local Interaction Score (LIS), Local Interaction Area (LIA), and ipTM scores are displayed for each interaction. Cut-off criteria to classify positive PPIs were set at LIS ≥ 0.203 and LIA ≥ 3432. Some interactions identified by AFM predictions were further experimentally validated through Co-IPs. These AFM predictions provided a preliminary model for the Zrf1 interaction network and guided the subsequent experimental validation of Zrf1-associated proteins (Fig 3C).
(TIF)

S4 Fig Validation of Zrf1 interactions and effects of RISC component knockdowns on GFP expression. (A) Schematic of the co-immunoprecipitation (Co-IP) workflow. GFP-tagged prey proteins were co-expressed with Zrf1-HA, followed by immunoprecipitation using α-HA magnetic beads. Immunoblots were used to detect GFP-tagged prey proteins, verifying interactions. (B–D) Co-IP experiments confirming interactions between Zrf1-HA and GFP-tagged RISC components (B) Zrf1-HA interacts with GFP-Fmr1, showing co-precipitation after immunoprecipitation with α-HA beads. mCherry-HA was used as a negative control. (C) Interaction between Zrf1-HA and GFP-Vig2a, with mCherry-HA as the control. (D) Co-IP showing that Zrf1-HA also interacts with GFP-Vig2b. Inputs verify protein expression levels, and controls demonstrate specificity of the Co-IP. (E) Immunoblots showing the specificity of antibodies against RISC components. RNAi-mediated knockdown of various RISC components on GFP expression over time. S2R+ cells were treated with dsRNA against *LacZ* (control), *GFP, Fmr1, vig, vig2, AGO2,* and *Tdrd3* for 0, 3, and 5 days. Western blots display levels of target proteins (e.g., Fmr1, Vig, Vig2, AGO2, Tdrd3) and GFP. Knockdown efficiency is confirmed by the reduction of the respective proteins, while α-Tubulin (αTub) serves as a loading control.
(TIF)

S5 Fig Protein-protein interaction of human ZRF1. (A) Schematic of the IP-MS workflow used to identify human ZRF1 interactors. HEK293T cells were transfected with mCherry-GFP, ZRF1-GFP, FLAG-mCherry, or FLAG-ZRF1. Immunoprecipitations (IPs) were performed using α-GFP or α-FLAG agarose/magnetic beads, followed by Coomassie staining and mass spectrometry analysis. (B) Coomassie-stained gel showing proteins immunoprecipitated using FLAG-tagged constructs (FLAG-mCherry, FLAG-ZRF1-stop). The FLAG-ZRF1-stop lane shows distinct protein bands, indicating specific enrichment of ZRF1-associated proteins. (C) Coomassie-stained gel displaying IP results for GFP-tagged constructs (mCherry-GFP, ZRF1-GFP). Bands in the ZRF1-GFP lane highlight the enrichment of ZRF1 and associated proteins. (D) Table summarizing the IP-MS spectral counts for human ZRF1 and known orthologs of *Drosophila* interactors. The table compares counts across different IP conditions (N-terminal vs. C-terminal tags) for FLAG-mCherry, FLAG-ZRF1, mCherry-GFP, and ZRF1-GFP, indicating consistency of interaction data across tagging strategies. Key interactors include FXR1, FXR2, DDX17, and SERBP1, which show orthology to *Drosophila* RISC components. (E) IPs with endogenous ZRF1 from HEK293T cells followed by immunoblotting for FMRP, FXR1, and FXR2, confirming interactions. Input lanes verify protein expression, while IgG serves as a negative control. (F) Co-IP experiments validating specific interactions between FLAG-ZRF1 and candidate interactors. Left: Interaction between FLAG-ZRF1 and SERBP1-GFP, and reciprocal IP using SERBP1-GFP and FLAG-ZRF1. Middle left: Co-IP showing binding between FLAG-ZRF1 and HABP4-GFP. Middle right: FLAG-ZRF1 precipitates with DDX17-Myc, DDX21-V5, and DDX5-GFP, confirming association. Bottom: GFP-tagged ZRF1 co-precipitates with FLAG-AGO1.
(TIF)

S6 Fig Complementation test and genetic interaction between *Zrf1* and *Pc.* (A) Table showing the results of complementation test between different *Zrf1* alleles and a deficiency line (*Df(3L)BSC553*) that covers the *Zrf1* locus. The number of non-TM (non-Tubby marker) offspring is listed against the total number of offspring, indicating whether the tested *Zrf1* alleles fail to complement each other or the deficiency. The absence of non-TM offspring suggests that the alleles are functionally compromised and do not complement, consistent with loss-of-function mutations. (B) Images of male forelegs (T1), midlegs (T2), and hindlegs (T3) from control, *Pc*^*1*^*/ + *heterozygous, and double heterozygous *Pc*^*1*^*/Zrf1* mutants (*Zrf1*^*148*^*, Zrf1*^*242*^). Control males have normal sex combs restricted to T1. *Pc*^*1*^/ + heterozygotes display ectopic sex combs on T2 and T3, and this phenotype is enhanced in *Pc*^*1*^*/Zrf1* double heterozygotes, indicating a genetic interaction between *Pc* and *Zrf1*.
(TIF)

S1 Data Source Data for all data presented in the figures are listed in the file. Fig 1A: Table A in S1 Data. Fig 1B: Table B in S1 Data. Fig 1C: Table C in S1 Data. Fig 1F: Table D in S1 Data. Fig 1K: Table E in S1 Data. Fig 1L: Table F in S1 Data. S1D Fig: Table G in S1 Data. S1E Fig: Table H in S1 Data. Fig 2A: Table I in S1 Data. Fig 2B: Table J in S1 Data. Fig 2C: Table K in S1 Data. Fig 2D: Table L in S1 Data. Fig 2I: Table M in S1 Data. Fig 2J: Table N in S1 Data. Fig 2K: Table O in S1 Data. Fig 2L: Table P in S1 Data. Fig 2M: Table Q in S1 Data. Fig 2N: Table R in S1 Data. Fig 2O: Table S in S1 Data. Fig 2P: Table T in S1 Data. Fig 4B: Table U in S1 Data. Fig 4C: Table V in S1 Data. Fig 5B: Table W in S1 Data. Fig 5D: Table X in S1 Data. Fig 5F: Table Y in S1 Data.
(XLSX)

S1 File Fly genotypes. (DOCX)
