# Supplementary figures and images for: The epigenetic factor Zrf1 regulates intestinal stem cell proliferation during midgut regeneration
Source: PLoS Genet. 2025 Oct 27;21(10):e1011910. doi: 10.1371/journal.pgen.1011910 (PMC12574921; doi:10.1371/journal.pgen.1011910)

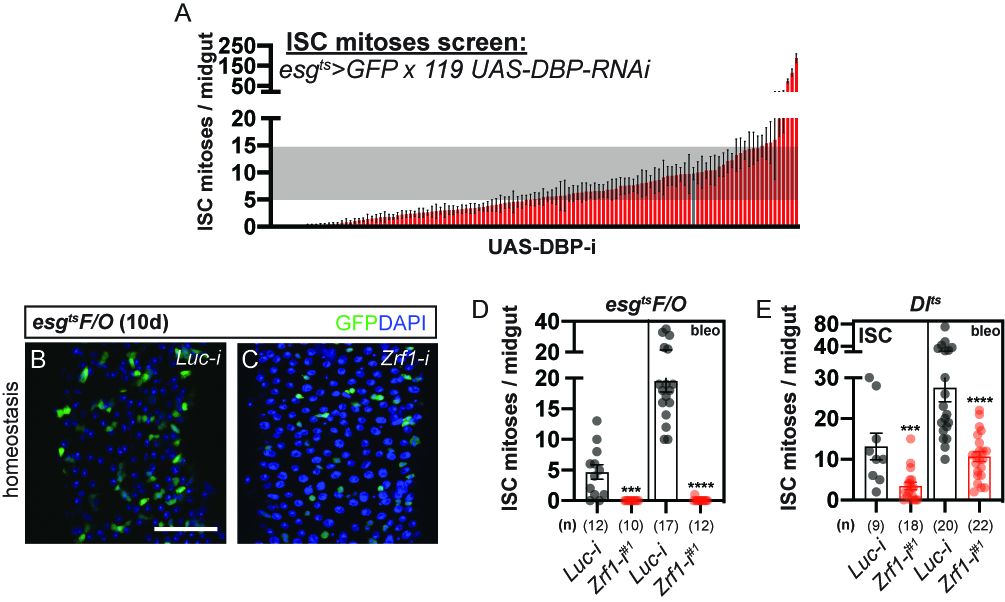

Supplement: S1 Fig — (A) ISC mitoses per midgut from an RNAi screen targeting 119 DNA-binding proteins (UAS-DBP-RNAi) driven by egtts > GFP. Each bar represents ISC mitoses for a specific RNAi line after 10 days at the permissive temperature. Red bars indicate lines with significantly reduced ISC mitoses compared to control (Luc-i). The gray bar is the Control (EGT/Luc-i). Zrf1 RNAi (Zrf1-i) is among the top hits causing a strong reduction in ISC proliferation. (B–C) Confocal images showing GFP + ISC/EB progenitor cells after 10 days of RNAi induction in egttsF/O flies during homeostasis. (B) Control (Luc-i) showing normal ISC proliferation, and (C) Zrf1 knockdown (Zrf1-i) showing a marked reduction in GFP+ progenitor cells. Green: GFP; blue: DAPI. Scale bar: 50 µm. (D) Quantification of ISC mitoses per midgut from egttsF/O flies after 10 days of RNAi induction during homeostasis or bleomycin (bleo) treatment. (E) ISC mitoses per midgut from DIts flies where Zrf1 RNAi was specifically driven in ISCs. ISC proliferation is markedly reduced upon Zrf1 knockdown compared to control (Luc-i), both during homeostasis and after bleomycin treatment. Bars represent mean ± SEM; n = number of midguts analyzed. Statistical significance determined by Student’s t-test; ***p < 0.001, ****p < 0.0001.*. (TIF) [file pgen.1011910.s001.tif]

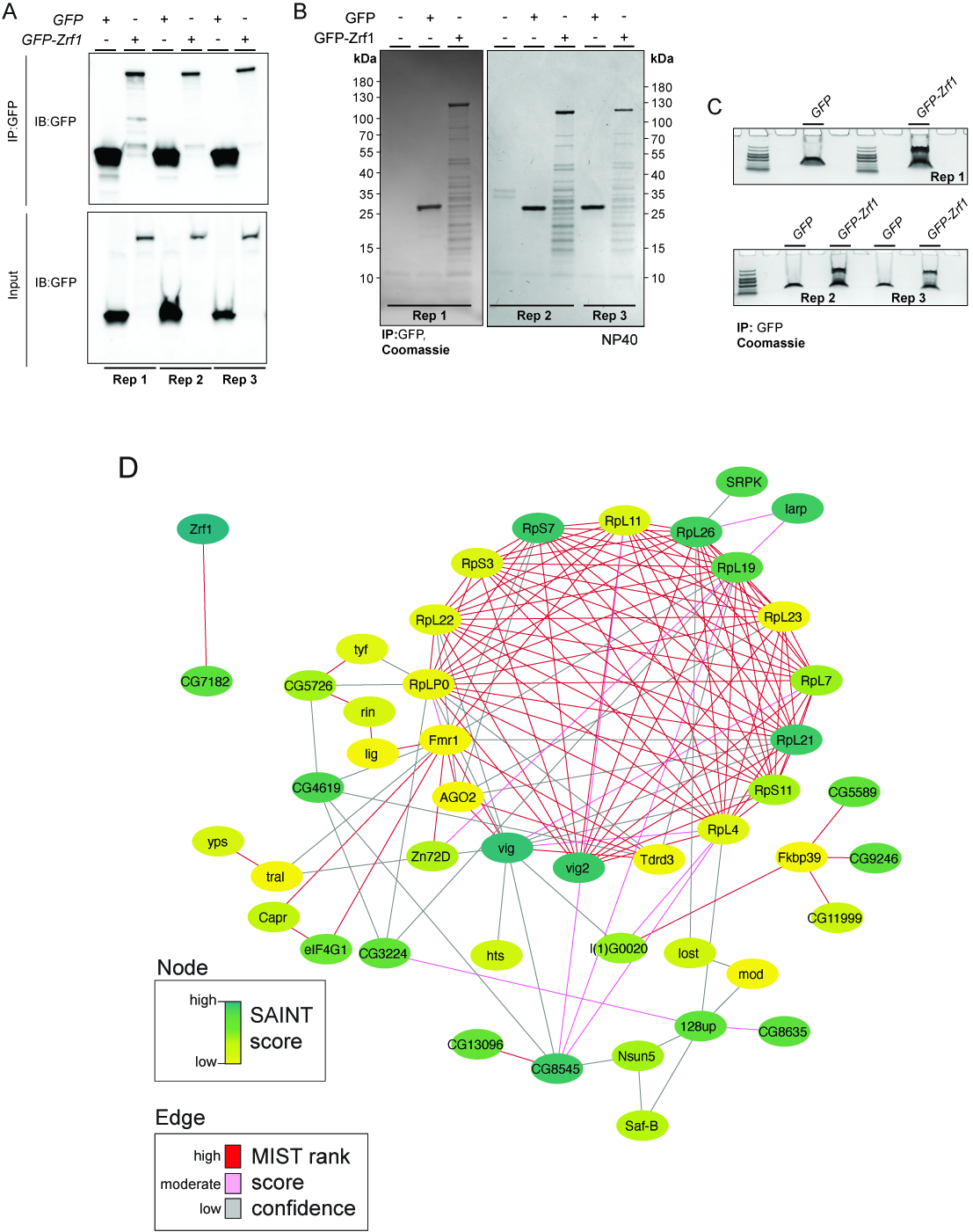

Supplement: S2 Fig — (A) Western blot analysis of immunoprecipitated (IP) samples from S2R+ cells expressing GFP or GFP-Zrf1 across three biological replicates (Rep 1, Rep 2, Rep 3). IP was performed using GFP-trap beads, and the presence of GFP-tagged proteins was confirmed by immunoblotting (IB) for GFP. Input samples show consistent expression levels across all replicates. (B) Coomassie-stained gels of IP samples from S2R+ cells expressing GFP or GFP-Zrf1. Three biological replicates (Rep 1, Rep 2, Rep 3) are shown, demonstrating consistent patterns of protein bands enriched in the GFP-Zrf1 lanes, confirming the reproducibility of Zrf1-associated protein complexes. Samples were prepared under mild detergent (NP40) conditions. (C) Additional Coomassie-stained gels highlighting the distinct protein bands observed in GFP-Zrf1 IP samples across the three replicates (Rep 1, Rep 2, Rep 3). This suggests specific enrichment of proteins that associate with Zrf1. (D) Network diagram showing the Zrf1 PPI network derived from IP-MS data. Nodes represent proteins, with colors indicating the SAINT score (green: high, yellow: low). Edges represent interactions, with line colors indicating MIST rank confidence (red: high, pink: moderate, gray: low). The network reveals associations between Zrf1 and multiple ribosomal proteins, RNA-binding proteins, and components of the RNA-induced silencing complex (RISC), highlighting the diverse interactome of Zrf1. IP-MS data validation was performed across three independent biological replicates, confirming reproducible interaction profiles. The network was constructed using SAINT and MIST scoring to assess interaction confidence. (TIF) [file pgen.1011910.s002.tif]

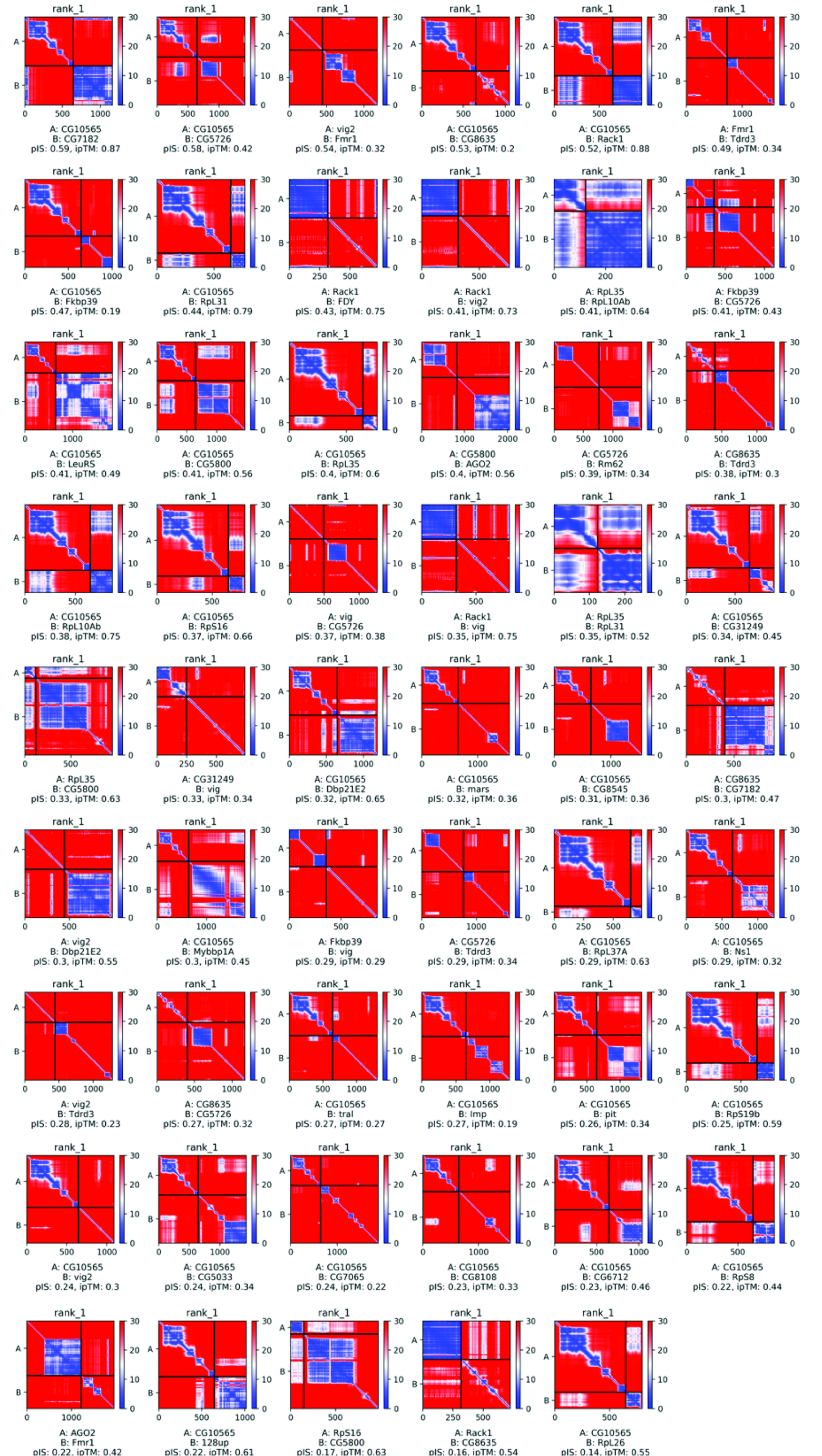

Supplement: S3 Fig — PAE maps of positive PPIs using AlphaFold-Multimer (AFM) screens to predict direct interactions between Zrf1 (CG10565) and candidate interactors identified from IP-MS data. Each PAE map represents the residue-residue alignment confidence between Zrf1 and a candidate interactor. In these PAE maps, blue regions indicate low predicted alignment error, suggesting high confidence in residue-residue alignment (potential interaction), while red regions denote high alignment error, indicating lower confidence. Local Interaction Score (LIS), Local Interaction Area (LIA), and ipTM scores are displayed for each interaction. Cut-off criteria to classify positive PPIs were set at LIS ≥ 0.203 and LIA ≥ 3432. Some interactions identified by AFM predictions were further experimentally validated through Co-IPs. These AFM predictions provided a preliminary model for the Zrf1 interaction network and guided the subsequent experimental validation of Zrf1-associated proteins (Fig 3C). (TIF) [file pgen.1011910.s003.tif]

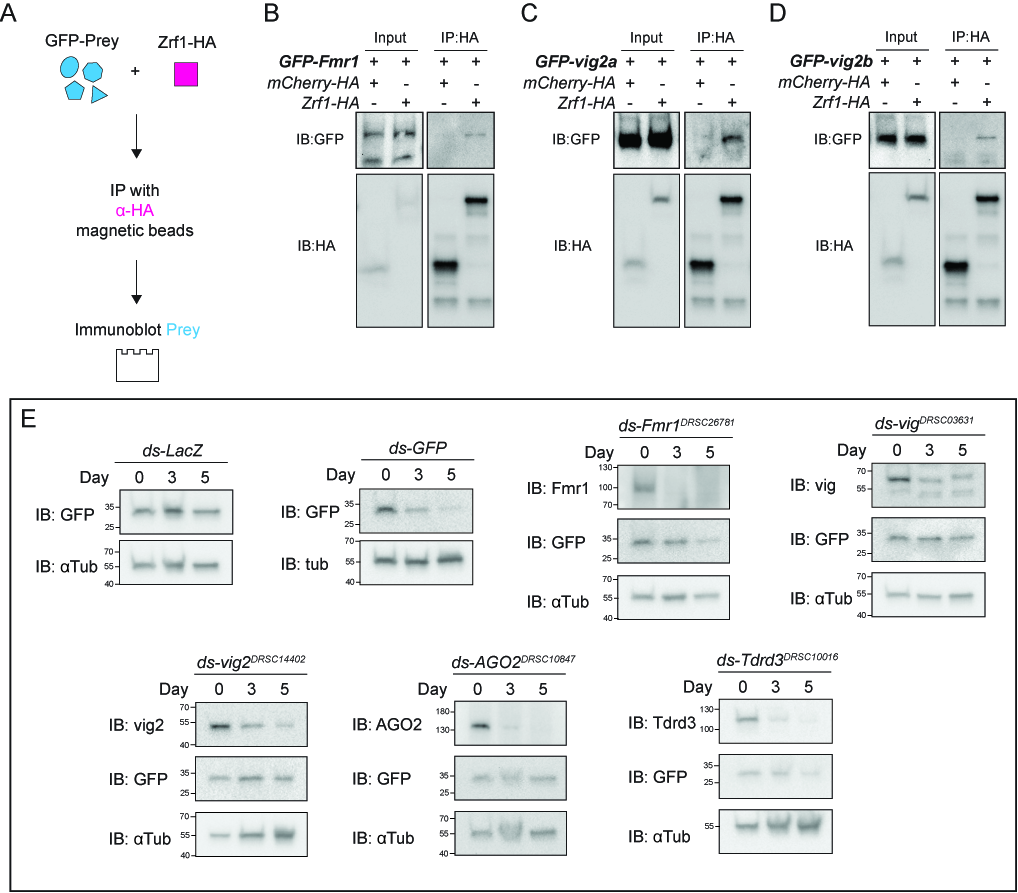

Supplement: S4 Fig — (A) Schematic of the co-immunoprecipitation (Co-IP) workflow. GFP-tagged prey proteins were co-expressed with Zrf1-HA, followed by immunoprecipitation using α-HA magnetic beads. Immunoblots were used to detect GFP-tagged prey proteins, verifying interactions. (B–D) Co-IP experiments confirming interactions between Zrf1-HA and GFP-tagged RISC components (B) Zrf1-HA interacts with GFP-Fmr1, showing co-precipitation after immunoprecipitation with α-HA beads. mCherry-HA was used as a negative control. (C) Interaction between Zrf1-HA and GFP-Vig2a, with mCherry-HA as the control. (D) Co-IP showing that Zrf1-HA also interacts with GFP-Vig2b. Inputs verify protein expression levels, and controls demonstrate specificity of the Co-IP. (E) Immunoblots showing the specificity of antibodies against RISC components. RNAi-mediated knockdown of various RISC components on GFP expression over time. S2R+ cells were treated with dsRNA against LacZ (control), GFP, Fmr1, vig, vig2, AGO2, and Tdrd3 for 0, 3, and 5 days. Western blots display levels of target proteins (e.g., Fmr1, Vig, Vig2, AGO2, Tdrd3) and GFP. Knockdown efficiency is confirmed by the reduction of the respective proteins, while α-Tubulin (αTub) serves as a loading control. (TIF) [file pgen.1011910.s004.tif]

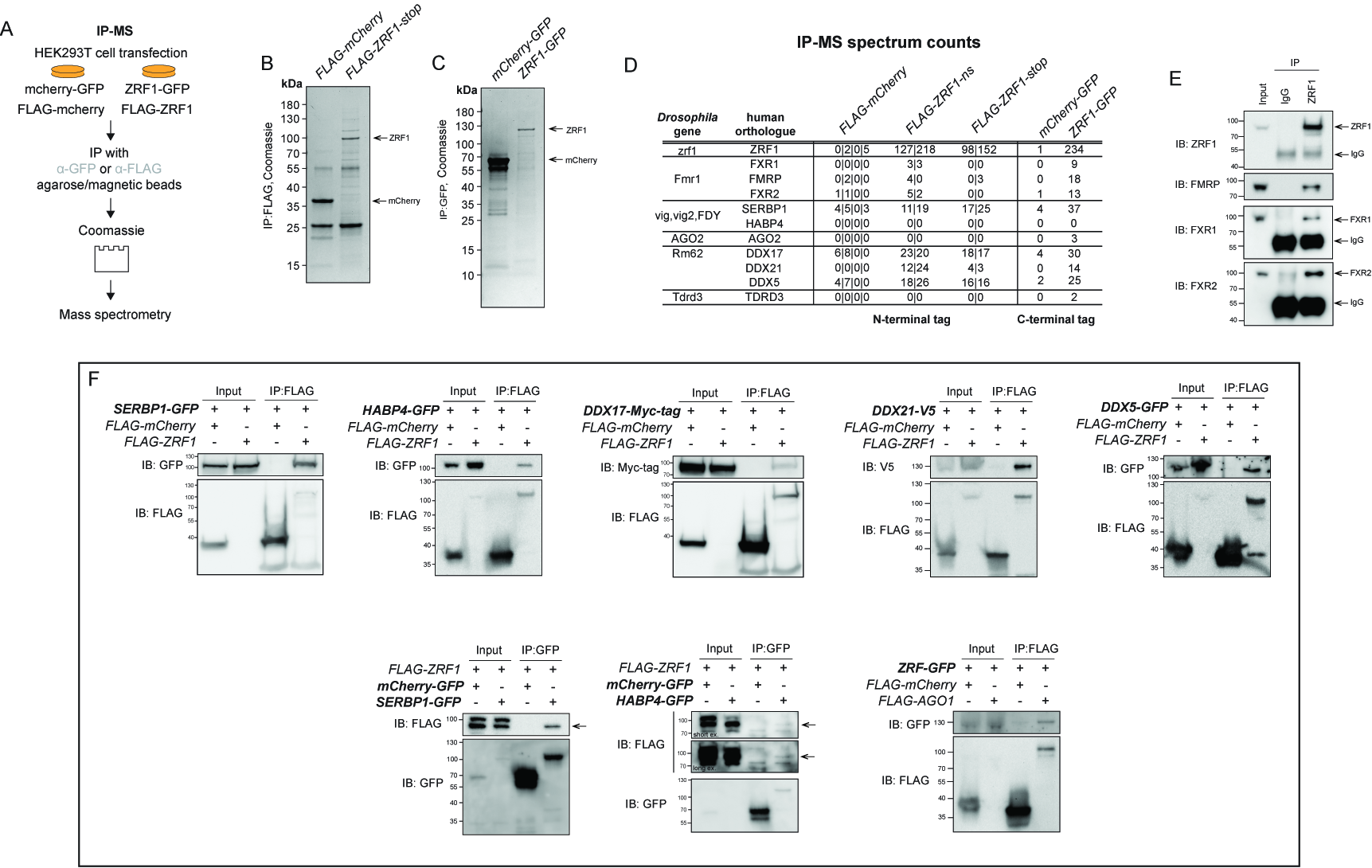

Supplement: S5 Fig — (A) Schematic of the IP-MS workflow used to identify human ZRF1 interactors. HEK293T cells were transfected with mCherry-GFP, ZRF1-GFP, FLAG-mCherry, or FLAG-ZRF1. Immunoprecipitations (IPs) were performed using α-GFP or α-FLAG agarose/magnetic beads, followed by Coomassie staining and mass spectrometry analysis. (B) Coomassie-stained gel showing proteins immunoprecipitated using FLAG-tagged constructs (FLAG-mCherry, FLAG-ZRF1-stop). The FLAG-ZRF1-stop lane shows distinct protein bands, indicating specific enrichment of ZRF1-associated proteins. (C) Coomassie-stained gel displaying IP results for GFP-tagged constructs (mCherry-GFP, ZRF1-GFP). Bands in the ZRF1-GFP lane highlight the enrichment of ZRF1 and associated proteins. (D) Table summarizing the IP-MS spectral counts for human ZRF1 and known orthologs of Drosophila interactors. The table compares counts across different IP conditions (N-terminal vs. C-terminal tags) for FLAG-mCherry, FLAG-ZRF1, mCherry-GFP, and ZRF1-GFP, indicating consistency of interaction data across tagging strategies. Key interactors include FXR1, FXR2, DDX17, and SERBP1, which show orthology to Drosophila RISC components. (E) IPs with endogenous ZRF1 from HEK293T cells followed by immunoblotting for FMRP, FXR1, and FXR2, confirming interactions. Input lanes verify protein expression, while IgG serves as a negative control. (F) Co-IP experiments validating specific interactions between FLAG-ZRF1 and candidate interactors. Left: Interaction between FLAG-ZRF1 and SERBP1-GFP, and reciprocal IP using SERBP1-GFP and FLAG-ZRF1. Middle left: Co-IP showing binding between FLAG-ZRF1 and HABP4-GFP. Middle right: FLAG-ZRF1 precipitates with DDX17-Myc, DDX21-V5, and DDX5-GFP, confirming association. Bottom: GFP-tagged ZRF1 co-precipitates with FLAG-AGO1. (TIF) [file pgen.1011910.s005.tif]

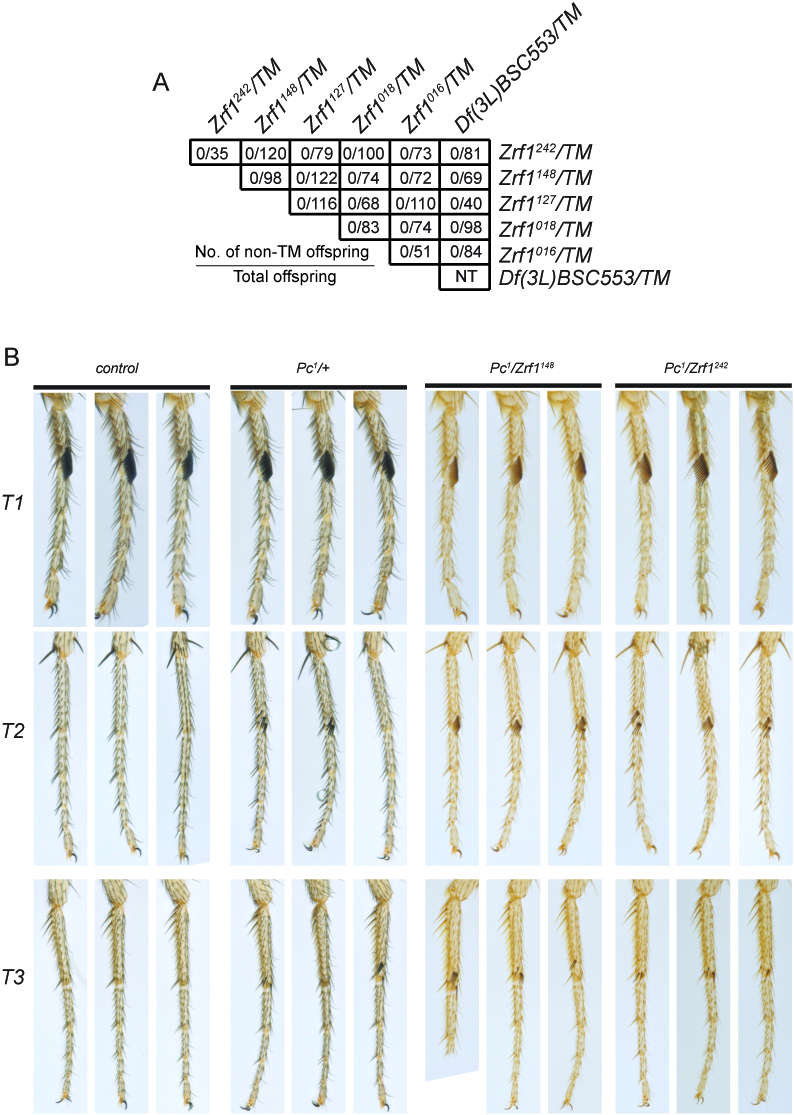

Supplement: S6 Fig — (A) Table showing the results of complementation test between different Zrf1 alleles and a deficiency line (Df(3L)BSC553) that covers the Zrf1 locus. The number of non-TM (non-Tubby marker) offspring is listed against the total number of offspring, indicating whether the tested Zrf1 alleles fail to complement each other or the deficiency. The absence of non-TM offspring suggests that the alleles are functionally compromised and do not complement, consistent with loss-of-function mutations. (B) Images of male forelegs (T1), midlegs (T2), and hindlegs (T3) from control, Pc1/ + heterozygous, and double heterozygous Pc1/Zrf1 mutants (Zrf1148, Zrf1242). Control males have normal sex combs restricted to T1. Pc1/ + heterozygotes display ectopic sex combs on T2 and T3, and this phenotype is enhanced in Pc1/Zrf1 double heterozygotes, indicating a genetic interaction between Pc and Zrf1. (TIF) [file pgen.1011910.s006.tif]
