# Supplementary material for: The epigenetic factor Zrf1 regulates intestinal stem cell proliferation during midgut regeneration
Source: PLoS Genet. 2025 Oct 27;21(10):e1011910. doi: 10.1371/journal.pgen.1011910 (PMC12574921; doi:10.1371/journal.pgen.1011910)
Supplement: S1 File — (DOCX) [file pgen.1011910.s008.docx]

**Fly genotypes**

**Fig 1:**

(B, C and J)

attP^VIE-260B^: *w*; *esg-Gal4, UAS-GFP, tub-Gal80^ts^/VIE-260b (KK control); +/+*

attP40: *w*; *esg-Gal4, UAS-GFP, tub-Gal80^ts^/attP40 (empty); +/+*

Luc-i: *w*; *esg-Gal4, UAS-GFP, tub-Gal80^ts^/+; UAS-Luc-i (JF01355)/+*

Zrf1-i #1: *w*; *esg-Gal4, UAS-GFP, tub-Gal80^ts^/UAS-Zrf1-i (HMC05212); +/+*

Zrf1-i #2: *w*; *esg-Gal4, UAS-GFP, tub-Gal80^ts^/UAS-Zrf1-i (GL01550); +/+*

Zrf1-i #3: *w*; *esg-Gal4, UAS-GFP, tub-Gal80^ts^/+; UAS-Zrf1-i (10565R-1)/+*

Zrf1-i #4: *w*; *esg-Gal4, UAS-GFP, tub-Gal80^ts^/UAS-Zrf1-i (KK102408); +/+*

Zrf1-i #5: *w*; *esg-Gal4, UAS-GFP, tub-Gal80^ts^/+; UAS-Zrf1-i (GD7019)/+*

p35: *w*; *esg-Gal4, UAS-GFP, tub-Gal80^ts^/+; UAS-p35/+*

Zrf1-i #1, p35: *w*; *esg-Gal4, UAS-GFP, tub-Gal80^ts^/UAS-Zrf1-i (HMC05212); UAS-p35/+*

DIAP: *w*; *esg-Gal4, UAS-GFP, tub-Gal80^ts^/+; UAS-DIAP/+*

Zrf1-i #1, DIAP: *w*; *esg-Gal4, UAS-GFP, tub-Gal80^ts^/UAS-Zrf1-i (HMC05212); UAS-DIAP/+*

(D and K) *w*; *esg-Gal4, UAS-GFP, tub-Gal80^ts^/+; UAS-Luc-i (JF01355)/Dl-LacZ*

(E and K) *w*; *esg-Gal4, UAS-GFP, tub-Gal80^ts^/UAS-Zrf1-i (HMC05212); Dl-LacZ/+*

(F and J) *w*; *esg-Gal4, UAS-GFP, tub-Gal80^ts^/+; UAS-FLP, act>CD2>Gal4/UAS-Luc-i (JF01355)*

(G and J) *w*; *esg-Gal4, UAS-GFP, tub-Gal80^ts^/UAS-Zrf1-i (HMC05212); UAS-FLP, act>CD2>Gal4/+*

(H and J) *w*; *esg-Gal4, UAS-GFP, tub-Gal80^ts^/UAS-sgZrf1 (CFD00331); UAS-Cas9.P2/+*

(I and J) *w*; *esg-Gal4, UAS-GFP, tub-Gal80^ts^/UAS-sgIntergenic (BEC); UAS-Cas9.P2/+*

(L) [from left to right]^28^

*w*; *esg-Gal4, UAS-GFP, tub-Gal80^ts^, Su(H)-Gal80/+; UAS-Luc-i (JF01355)/+*

*w*; *esg-Gal4, UAS-GFP, tub-Gal80^ts^, Su(H)-Gal80/UAS-Zrf1-i (HMC05212); +/+*

*w*; *Su(H)GBE-Gal4/+; tub-Gal80^ts^/UAS-Luc-i (JF01355)*

*w*; *Su(H)GBE-Gal4/UAS-Zrf1-i (HMC05212); tub-Gal80^ts^/+*

*w*; *tub-Gal80^ts^/+; pros-Gal4/UAS-Luc-i (JF01355)*

*w*; *tub-Gal80^ts^/UAS-Zrf1-i (HMC05212); pros-Gal4/+*

**Fig 2:**

(A and B)

w^1118^: *w*; *esg-Gal4, UAS-GFP, tub-Gal80^ts^/+; +/+* (*egt^ts^* crossed with *w^1118^*)

Zrf1^P{EP}G4964^: *w*; *esg-Gal4, UAS-GFP, tub-Gal80^ts^/+; Zrf1^P{EP}G4964^/+*

Zrf1^P{EPgy2}G4964^: *w*; *esg-Gal4, UAS-GFP, tub-Gal80^ts^/+; Zrf1^P{EPgy2}G4964^/+*

Zrf1^ORF.3xHA^: *w*; *esg-Gal4, UAS-GFP, tub-Gal80^ts^/+; Zrf1^ORF.3xHA^/+*

Zrf1^ORF.CC^: *w*; *esg-Gal4, UAS-GFP, tub-Gal80^ts^/+; Zrf1^ORF.CC^/+*

(C and D)

attp40: *w*; *esg-Gal4, UAS-GFP, tub-Gal80^ts^/attP40 (empty); UAS-dCas9-VPR/+*

sg-Zrf1: *w*; *esg-Gal4, UAS-GFP, tub-Gal80^ts^/sg-Zrf1(GS03041); UAS-dCas9-VPR/+*

(E-I)

w^1118^: *w*; *esg-Gal4, UAS-GFP, tub-Gal80^ts^/+; +/+* (*egt^ts^* crossed with *w^1118^*)

Egfr^Top4.2^: *w/UAS-Egfr^Top4.2^*; *esg-Gal4, UAS-GFP, tub-Gal80^ts^/+; +/+*

Egfr^Top4.2^+Zrf1-i: *w/UAS-Egfr^Top4.2^*; *esg-Gal4, UAS-GFP, tub-Gal80^ts^/UAS-Zrf1-i (HMC05212); +/+*

Ras1^A^: *w*; *esg-Gal4, UAS-GFP, tub-Gal80^ts^/+; UAS-Ras1^A^/+*

Ras1^A^+Zrf1-i: *w*; *esg-Gal4, UAS-GFP, tub-Gal80^ts^/UAS-Zrf1-i (HMC05212); UAS-Ras1^A^/+*

Raf^F179^: *w*; *esg-Gal4, UAS-GFP, tub-Gal80^ts^/+; UAS-Raf^F179^/+*

Raf^F179^+Zrf1-i: *w*; *esg-Gal4, UAS-GFP, tub-Gal80^ts^/UAS-Zrf1-i (HMC05212); UAS-Raf^F179^/+*

hRaf^gof^: *w*; *esg-Gal4, UAS-GFP, tub-Gal80^ts^/+; UAS-hRaf^gof^/+*

hRaf^gof^+Zrf1-i: *w*; *esg-Gal4, UAS-GFP, tub-Gal80^ts^/+; UAS-hRaf^gof^/+*

(J)

Luc-i^JF01355^: *w*; *esg-Gal4, UAS-GFP, tub-Gal80^ts^/+; UAS-Luc-i (JF01355)/+*

N-i^HMS00009^: *w*; *esg-Gal4, UAS-GFP, tub-Gal80^ts^/+; UAS-N-i (HMS00009)/+*

N-i^HMS00009^+ Zrf-i: *w*; *esg-Gal4, UAS-GFP, tub-Gal80^ts^/UAS-Zrf1-i (HMC05212); UAS-N-i (HMS00009)/+*

N-i^JF01637^: *w*; *esg-Gal4, UAS-GFP, tub-Gal80^ts^/+; UAS-N-i (JF01637)/+*

N-i^JF01637^+Zrf1-i: *w*; *esg-Gal4, UAS-GFP, tub-Gal80^ts^/UAS-Zrf1-i (HMC05212); UAS-N-i (JF01637)/+*

N-i^HMS00001^: *w*; *esg-Gal4, UAS-GFP, tub-Gal80^ts^/+; UAS-N-i (HMS00001)/+*

N-i^HMS00001^+Zrf1-i: *w*; *esg-Gal4, UAS-GFP, tub-Gal80^ts^/UAS-Zrf1-i (HMC05212); UAS-Luc-i (HMS00001)/+*

(K)

w^1118^: *w*; *esg-Gal4, UAS-GFP, tub-Gal80^ts^/+; +/+* (*egt^ts^* crossed with *w^1118^*)

yki^WT^: *w*; *esg-Gal4, UAS-GFP, tub-Gal80^ts^/+; UAS-yki^WT^/+*

yki^WT^+Zrf1-i: *w*; *esg-Gal4, UAS-GFP, tub-Gal80^ts^/UAS-Zrf1-i (HMC05212); UAS-yki^WT^/+*

yki^3SA^: *w*; *esg-Gal4, UAS-GFP, tub-Gal80^ts^/+; UAS-yki^3SA^/+*

yki^3SA^+Zrf1: *w*; *esg-Gal4, UAS-GFP, tub-Gal80^ts^/UAS-Zrf1-i (HMC05212); UAS-yki^3SA^/+*

**Fig 4:**

Genotypes are as listed in the figure.

**Fig 5:**

(C and D)

y,w/Y: *y,w/Y*

OreR/Y: *OreR/Y*

Control: *w^m4h^,ac^1^,y^1^/Y* (*w^m4h^,ac^1^,y^1^* females crossed with *y,w* males)

Fmr1^d50M^/+: *w^m4h^,ac^1^,y^1^/Y; +/+; Fmr1^d50M^/+*

Fmr1^d113M^/+: *w^m4h^,ac^1^,y^1^/Y: +/+: Fmr1^d113M^/+*

vig^C274^/+: *w^m4h^,ac^1^,y^1^/Y; vig^C274^/+; +/+*

vig^d65-72^/+: *w^m4h^,ac^1^,y^1^/Y;* vig^d65-72^/+; +/+

vig^d63-73^/+: *w^m4h^,ac^1^,y^1^/Y; vig^d63-73^/+; +/+*

vig2^PL00470^/+: *w^m4h^,ac^1^,y^1^/Y; +/+; vig2^PL00470^/+*

vig2^Gd59-75^/+: *w^m4h^,ac^1^,y^1^/Y; +/+; vig2^Gd59-75^/+*

FDY^WT_E5^: *w^m4h^,ac^1^,y^1^/Y, FDY^WT_E5^* (control for FDY mutants)

FDY^MUT_L3^: *w^m4h^,ac^1^,y^1^/Y, FDY^MUT_L3^*

FDY^MUT_L64^: *w^m4h^,ac^1^,y^1^/Y, FDY^MUT_L64^*

AGO2^414^/+: *w^m4h^,ac^1^,y^1^/Y; +/+; AGO2^414^/+*

Rm62^01086^/+: *w^m4h^,ac^1^,y^1^/Y: +/+; Rm62^01086^/+*

BSC553/+: *w^m4h^,ac^1^,y^1^/Y: +/+; BSC553/+*

Zrf1^G4964^/+: *w^m4h^,ac^1^,y^1^/Y: +/+; Zrf1^G4964^/+*

Zrf1^cr01803^/+: *w^m4h^,ac^1^,y^1^/Y: +/+; Zrf1^cr01803^/+*

Zrf1^016^/+: *w^m4h^,ac^1^,y^1^/Y: +/+; Zrf1^016^/+*

Zrf1^018^/+: *w^m4h^,ac^1^,y^1^/Y: +/+; Zrf1^018^/+*

Zrf1^127^/+: *w^m4h^,ac^1^,y^1^/Y: +/+; Zrf1^127^/+*

Zrf1^148^/+: *w^m4h^,ac^1^,y^1^/Y: +/+; Zrf1^148^/+*

Zrf1^242^/+: *w^m4h^,ac^1^,y^1^/Y: +/+; Zrf1^242^/+*

(E and F)

Genotypes are as listed in the figure.

**S1** Fig**:**

(B, C and D)

Luc-i: *w*; *esg-Gal4, UAS-GFP, tub-Gal80^ts^/+; UAS-FLP, act>CD2>Gal4/UAS-Luc-i (JF01355)*

Zrf1-i: *w*; *esg-Gal4, UAS-GFP, tub-Gal80^ts^/UAS-Zrf1-i (HMC05212); UAS-FLP, act>CD2>Gal4/+*

(E)

Luc-i: *w*; *tub-Gal80^ts^/+; Dl-Gal4/UAS-Luc-i (JF01355)*

Zrf1-i: *w*; *tub-Gal80^ts^/UAS-Zrf1-i (HMC05212); Dl-Gal4/+*

**S6** Fig**:**

Genotypes are as listed in the figure.
